# Cyanobacterial Diversity in Microbial Mats from the Hypersaline Lagoon System of Araruama, Brazil: An In-depth Polyphasic Study

**DOI:** 10.3389/fmicb.2017.01233

**Published:** 2017-06-30

**Authors:** Vitor M. C. Ramos, Raquel Castelo-Branco, Pedro N. Leão, Joana Martins, Sinda Carvalhal-Gomes, Frederico Sobrinho da Silva, João G. Mendonça Filho, Vitor M. Vasconcelos

**Affiliations:** ^1^Faculty of Sciences, University of PortoPorto, Portugal; ^2^Interdisciplinary Centre of Marine and Environmental Research (CIIMAR/CIMAR), University of PortoMatosinhos, Portugal; ^3^Palynofacies and Organic Facies Laboratory, Department of Geology, Federal University of Rio de JaneiroRio de Janeiro, Brazil

**Keywords:** cyanobacteria, microbial mats, hypersaline, diversity, morphological characterization, 16S rRNA gene, phylogeny, next-generation sequencing

## Abstract

Microbial mats are complex, micro-scale ecosystems that can be found in a wide range of environments. In the top layer of photosynthetic mats from hypersaline environments, a large diversity of cyanobacteria typically predominates. With the aim of strengthening the knowledge on the cyanobacterial diversity present in the coastal lagoon system of Araruama (state of Rio de Janeiro, Brazil), we have characterized three mat samples by means of a polyphasic approach. We have used morphological and molecular data obtained by culture-dependent and -independent methods. Moreover, we have compared different classification methodologies and discussed the outcomes, challenges, and pitfalls of these methods. Overall, we show that Araruama's lagoons harbor a high cyanobacterial diversity. Thirty-six unique morphospecies could be differentiated, which increases by more than 15% the number of morphospecies and genera already reported for the entire Araruama system. Morphology-based data were compared with the 16S rRNA gene phylogeny derived from isolate sequences and environmental sequences obtained by PCR-DGGE and pyrosequencing. Most of the 48 phylotypes could be associated with the observed morphospecies at the order level. More than one third of the sequences demonstrated to be closely affiliated (best BLAST hit results of ≥99%) with cyanobacteria from ecologically similar habitats. Some sequences had no close relatives in the public databases, including one from an isolate, being placed as “loner” sequences within different orders. This hints at hidden cyanobacterial diversity in the mats of the Araruama system, while reinforcing the relevance of using complementary approaches to study cyanobacterial diversity.

## Introduction

Photosynthetic microbial mats are complex, micro-scale ecosystems that can be found globally in a wide range of environments and are a major driving force in the formation of some modern microbialites, i.e., biologically-induced mineralization driven by microbial growth (Dupraz et al., 2009; Decho, 2010; Stal, 2012). Photosynthetic mats usually have an upper green layer where cyanobacteria predominate (Ward et al., 2006; Dupraz et al., 2009; Stal, 2012). This layer is the most exposed in terms of environmental changes and disturbances, with cyanobacteria acting as primary producers (Ley et al., 2006; Stal, 2012; Harris et al., 2013) and atmospheric nitrogen fixers (Díez et al., 2007; Bauersachs et al., 2011; Stal, 2012), while being responsible for the production of a matrix of extracellular polymeric substances that provide physical protection and resistance to desiccation for the microbial mat community (Dupraz et al., 2009; Franks and Stolz, 2009; Stal, 2012).

In saline aquatic systems, these photosynthetic mats can be observed in flat, undisturbed, sheltered marine or estuarine coasts, in salterns or salt evaporation ponds, or in hypersaline lagoons (Oren, 2012; Stal, 2012). In hypersaline lagoon margins, where photosynthetic mats develop, high salinity, seasonal desiccation, and high solar irradiance are the main environmental stressors influencing microbial mat community adaptation processes (Stal, 2012). For instance, salinity levels determine that only halophilic or halotolerant cyanobacteria are able to inhabit and appropriately develop in these environments. Some studies have listed taxa commonly occurring at high salt concentrations (e.g., see Oren, 2012). However, due to taxonomy-related issues, obtaining an accurate and comprehensive list of cyanobacterial species is challenging (Oren, 2012; Dvorák et al., 2015; Komárek, 2016). Traditional systems of classification and identification keys of cyanobacteria are based mainly on morphological criteria (for a review, see Komárek et al., 2014), something that is now recognized as not only lacking taxonomic resolution when applying the more recent cyanobacterial species concepts, but also to completely ignore cryptic species (Dvorák et al., 2015). The latter, of which numerous examples are known, correspond to morphologically indistinguishable cyanobacteria that do not share a common evolutionary history (Komárek et al., 2014; Dvorák et al., 2015; Komárek, 2016). On the other hand, public databases (e.g., GenBank, SILVA, Greengenes, RDP) feature a large number of misidentified cyanobacterial sequences (Komárek, 2016). Also, sequences from reference strains that cover all known cyanobacterial diversity are still missing in such databases (Garcia-Etxebarria et al., 2014; Tuzhikov et al., 2014; Komárek, 2016). Therefore, a polyphasic approach that includes molecular, morphological and ecophysiological traits is now mandatory for the taxonomy and identification of cyanobacteria (Komárek et al., 2014; Komárek, 2016).

The east coastline region of the State of Rio de Janeiro in Brazil harbors a series of shallow coastal lagoons, forming one of the major hypersaline systems of the world (Clementino et al., 2008). The main waterbody is the Araruama lagoon, which lends its name to the system and has a remarkably high salt content (average of 5.2% total salts, Clementino et al., 2008). This region has a typical tropical climate with wet and dry seasons, where low levels of annual rainfall and high evaporation rates favor the development of several salty ponds around the lagoons (Kjerfve et al., 1996; Clementino et al., 2008). The cyanobacterial species present in microbial mats and/or water samples from several lagoons of the Araruama system have been extensively studied through culture-independent, morphological-based identifications (e.g., Iespa and Silva, 2005; Silva et al., 2006, 2007a,b, 2011). These studies show that Araruama's lagoons harbor a high diversity of cyanobacteria. By contrast, a single molecular-based study of the total microbial diversity has been performed by Clementino et al. (2008), using water samples from the Araruama lagoon. These authors have only detected three cyanobacterial phylotypes, *Coleofasciculus chthonoplastes, Halothece* sp., and *Synechococcus* sp.

With the aim of strengthening the knowledge on the cyanobacterial diversity present in the Araruama's complex by (1) considering the existing morphological-based species inventories, (2) taking into account the findings from Mobberley et al. (2012) and Harris et al. (2013), who demonstrate the power of 454 sequencing technology for the study of the microbial diversity in very complex samples such as photosynthetic hypersaline mats, (3) realizing that an accurate identification may be hampered by low resolution of classification methods (Dvorák et al., 2015; Nguyen et al., 2016), and (4) following the more recent principles and recommendations for studying cyanobacterial taxa (Dvorák et al., 2015; Komárek, 2016), we have characterized the cyanobacteria present in three mats from three lagoons of the Araruma system. For this purpose, we have followed a polyphasic approach combining culture-dependent and -independent techniques, and in order to understand how distinct definitions of “units of diversity” may shape the perceived cyanobacterial community structure of the mats (composition, richness, and diversity), we have compared different classification methods for the sequences.

## Materials and methods

### Sampling sites

**Figure 1:**
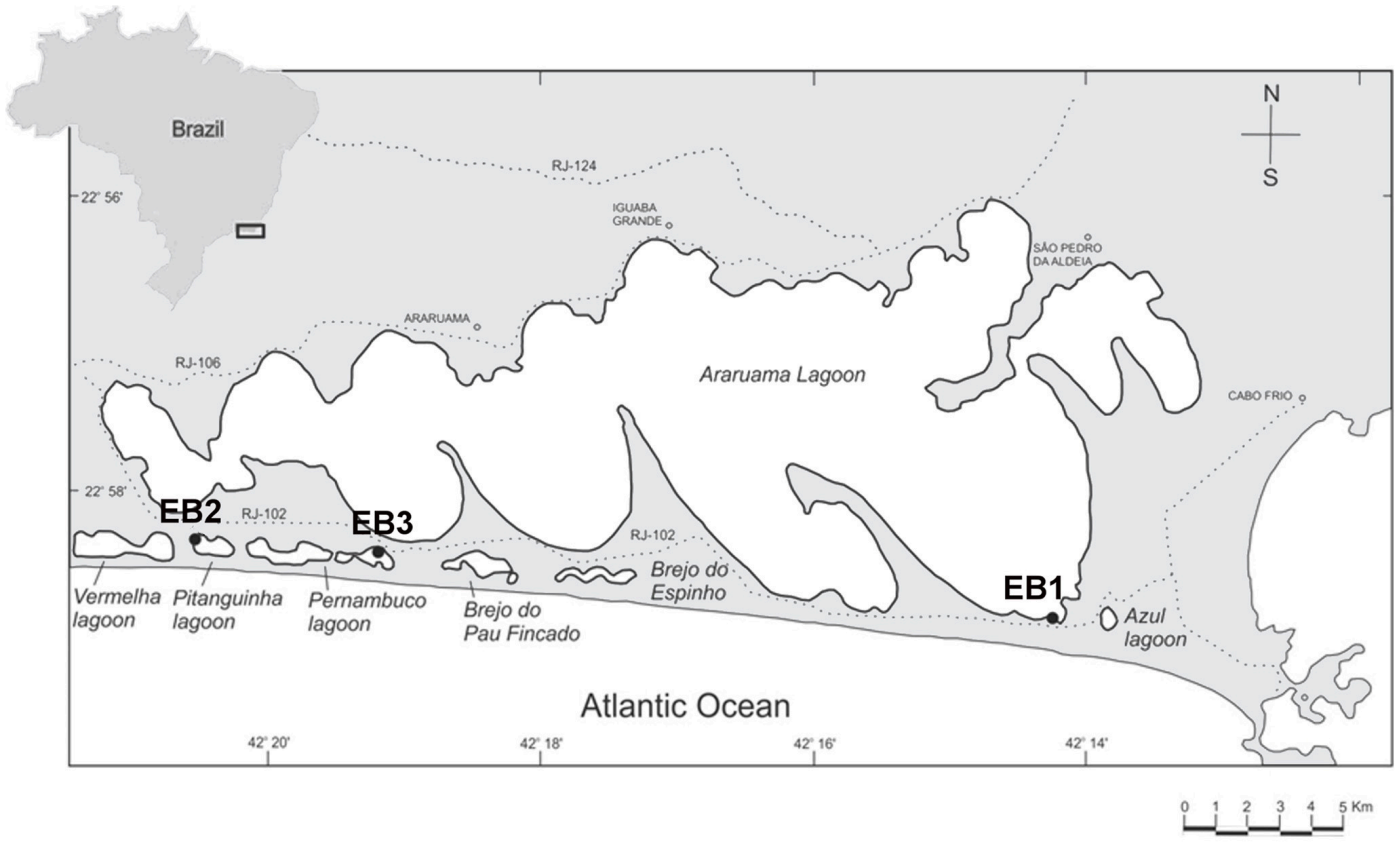
Araruama complex and sampling sites. EB1 is located in the main lagoon (Araruama), EB2 in lagoon Pitanguinha, and EB3 in lagoon Pernambuco.

Samples were collected from three lagoons of the Araruama complex (Figure 1): Araruama (the main lagoon; 22°56′36.0″S 42°06′02.0″W), Pitanguinha (22°55′39.0″S 42°21′20.0″W), and Pernambuco (22°55′50.0″S 42°18′86.0″W). Sampling sites (EB1, EB2, and EB3, respectively) were selected based on the occurrence of cyanobacterial-dominated mats, as previously indicated in Damazio and Silva (2006), Iespa and Silva (2005), and Silva et al. (2005, 2006). EB1 is placed in the eastern part of Araruama, at the entry of a confined, temporary pond connected to the main lagoon by a small channel (Supplementary Image S1). The site is near the Channel Itajuru in Cabo Frio (Figure 1), which connects the lagoon Araruama with the Atlantic Ocean. EB2 is surrounded by typical restinga vegetation (Supplementary Image S1) and is located near to a salt pan. EB3 is located in an artificial pond surrounded by grass-like vegetation, adjacent to the lagoon, and is also near a salt pan.

### Field sampling, sample processing, and study design

Mat samples from each site were collected from an area of 1 m^2^ (Supplementary Image S1D), in February during the rainy season (Supplementary Images S1D–F). Mats from these sampled areas were macroscopically homogeneous. Physicochemical parameters of the water above or near the sampled mats were determined and are presented in Table 1. The shape of the mats was recorded during sampling, while their structural characteristics were examined at the laboratory. For this purpose, mat sections of about 10 × 10 cm (Supplementary Images S1G–I) were collected, stored into polypropylene bags and transported to the lab. Mats were then characterized by color and carbonate lamination under a light stereoscopic microscope.

**Table 1:** Physicochemical parameters^*^ of the three studied sites located in the Araruama lagoon complex.

**Sampling site**	**Lagoon**	**pH**	**Conductivity (μS/cm)**	**Salinity (psu)**	**Water temperature (°C)**	**Air temperature (°C)**
EB1	Araruama	8.3 (8.5)	24.2	(35.3)	24 (30.1)	27 (27.5)
EB2	Pitanguinha	8.4 (8.6)	9.2	(56.9)	34 (32.1)	31 (28.9)
EB3	Pernambuco	7.7 (8.8)	11.2	(59.8)	28 (31.5)	27 (28.8)

* *At the time of sampling. Within parentheses are average values for the 13 months prior to sampling*.

Subsamples used for isolation and morphological and molecular characterizations of cyanobacteria present in the mats were separated just after sample collection. Sections of 2-cm diameter from the top layers of the mats were haphazardly collected within the sampled area using a polypropylene sampler and distributed into 50 ml falcon sterile tubes. Subsamples were transported and preserved in the dark at 4°C. Soon arriving at the laboratory, they were processed aseptically and carefully restricted to their top photosynthetic layer (< 3.5 mm; see the Results Section), using sterile scalpel blades. All subsamples were screened for the presence of cyanobacteria by observing a piece of the mat under a light microscope (Leica DMLB, Bensheim, Germany).

**Figure 2:**
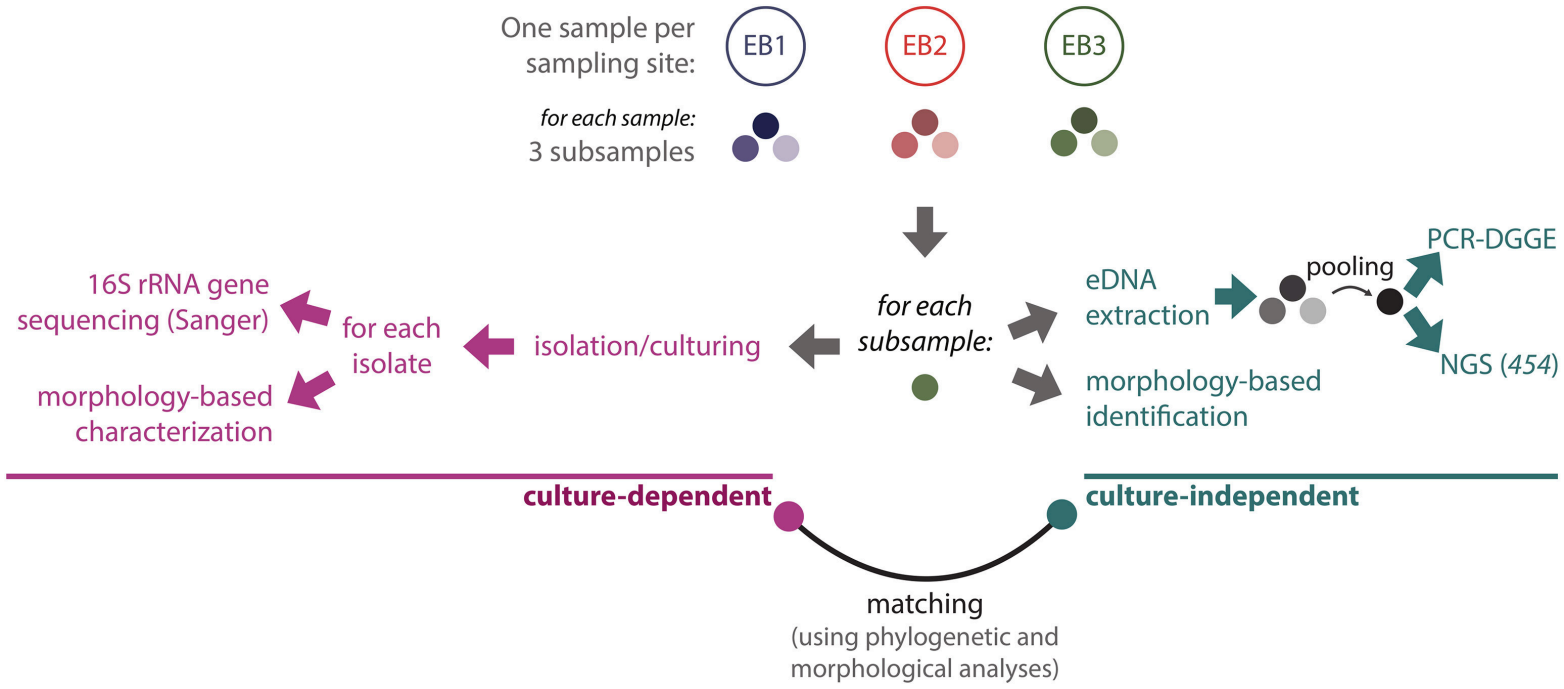
Schematic overview of the experimental design. See text for methodological details.

A workflow diagram illustrating the experimental procedures used in this polyphasic study is shown in Figure 2. For each mat sample, three subsamples were independently used in each methodological approach. For instance, three independent slide preparations were observed for the microscope-based characterization of each environmental sample. The same applies for the isolation of cyanobacteria and of environmental DNA.

### Isolation, culturing, and morphological-based characterization of cyanobacteria

For the isolation of cyanobacteria, subsamples were subjected to liquid culture enrichment, streaking in agar plates or micromanipulation (Rippka, 1988; Waterbury, 2006; see also Brito et al., 2012), or to a combination thereof, using different cultures media and salinities. Whenever feasible (i.e., for dominant species) single cells, colonies, or filaments were isolated under the microscope with the help of a stretched Pasteur pipette, and transferred directly from raw biological material to different liquid or solid media (Ramos et al., 2010). When growth was evident, aliquots from the enriched cultures or agar plates were transferred and streaked again into fresh agar plates, or isolated by micromanipulation. The process was repeated until unicyanobacterial cultures were obtained. The non-axenic isolates were then transferred and grown in the correspondent liquid medium. The different media used during isolation were MN, BG11_0_, and Z8, at NaCl concentrations of 25, 40, or 55 o (Rippka, 1988; Waterbury, 2006) and were supplemented with B_12_ vitamin and cycloheximide (Rippka, 1988). During the isolation process, cultures were kept under a light/dark regime of 14:10 h, irradiance of 10–30 μmol photons m^−2^ s^−1^, and temperature of 25°C. Isolates were deposited at the Blue Biotechnology and Ecotoxicology Culture Collection (acronym LEGE), at CIIMAR, Matosinhos, Portugal.

Microphotographs of environmental samples and isolates (either bright field or fluorescence) were obtained using a microscope (Model BX41, Olympus, Hamburg, Germany) coupled to an image analysis system (Model DP72 microscope digital camera, Olympus). Filament and/or cell dimensions were measured using the software Cell B (Olympus), with the same equipment. Dominant or abundant species (qualitative measure) present in each mat sample were recorded.

### Survey of cyanobacterial taxa from previous publications

A primary literature search was performed to assess the cyanobacterial species richness previously recorded in the Araruama's complex. As a consequence, a checklist of taxa was created, which also includes the cyanobacterial taxa recorded in this study (Supplementary Table S1).

### DNA isolation and 16S rRNA gene amplification

For each sample, three microbial mat subsamples (Figure 2) were homogenized using sterile mortars and pestles. Approximately 400 mg (wet weight) of material was used for each DNA extraction. Total environmental DNA (eDNA) was extracted from samples using the Zymo Research Soil Microbe DNA kit (Zymo Research Corp, Irvine, CA, USA), according to the manufacturer's instructions. DNA integrity was checked by agarose gel electrophoresis with ethidium bromide staining. DNA concentration was determined and normalized between samples (and subsamples) as described in Leão et al. (2012), and then the triplicates were pooled (Figure 2). With respect to isolates, genomic DNA (gDNA) was extracted from fresh biomass samples, harvested from log-phase cultures, using the commercial kit PureLink™ Genomic DNA Mini Kit (Invitrogen, Carlsbad, USA).

In the case of gDNA from isolates, PCRs were performed using the conditions and the primer sets previously described in Brito et al. (2012). Regarding eDNA samples, a fragment of 422 bp length was amplified using the cyanobacteria-specific primer pair CYA-359F/CYA-781R (Nübel et al., 1997). In PCRs for denaturing gradient gel electrophoresis (DGGE) analysis, the forward primer (CYA-359F-GC) had a 40-nucleotide GC-rich sequence (GC clamp) attached to its 5′-end. The PCR reactions for DGGE were prepared in a volume of 20 μl containing 1× Reaction Buffer, 2.5 mM MgCl_2_, 200 μM of each deoxynucleotide triphosphate, 20.0 pmol of each primer, 0.5 U of GoTaq® Flexi DNA Polymerase (Promega, Madison, WI, USA), 20 mg ml^−1^ of bovine serum albumin (BSA), and 5–10 ng of DNA template. Thermal cycling was carried out in a T-Professional Standard thermocycler (Biometra, Goettingen, Germany) under the following conditions: initial denaturation at 94°C for 2 min, followed by 11 cycles at 94°C for 1 min, 65°C for 1 min, and 72°C for 1 min. This first step was followed by 32 cycles at 94°C for 1 min, 55°C for 1 min, and 72°C for 4 min and a final extension step at 72°C for 4 min. PCR products were separated by 1.5% (w/v) agarose gel in 1× TAE buffer (40 mM Tris, 20 mM acetic acid, 1 mM EDTA). Gels were stained with ethidium bromide and photographed under UV transillumination.

### Denaturing gradient gel electrophoresis, and cloning

After gel visualization, PCR products from the same mat sample were pooled. Twenty microliters of each pooled sample were loaded onto 6% polyacrylamide 1 mm gels, using a 40–60% denaturing gradient (100% denaturing conditions correspond to 7 M urea and 40% formamide). The electrophoresis was performed using a DCode system (Bio-Rad, CA, USA) at 60 V for 16 h, in 1× TAE buffer. The gel was stained with 1× SYBR Gold nucleic acid stain (Invitrogen, San Diego, CA). Small pieces of visible DGGE bands (Supplementary Image S2) were punched from the gel with sterile pipette tips. Each piece was then transferred into PCR tubes containing 30 μl of sterile water and incubated at 37°C for 30 min to allow diffusion of the DNA. Two microliters of the eluted DNA were used as template for the re-amplification of the 16S rRNA gene, as described above. In this case, CYA-359F (i.e., without the GC clamp) was the forward primer used, as described by Nübel et al. (1997). PCR products were then extracted from the agarose gel and purified by using the spin columns Cut & Spin Gel Extraction (GRiSP, Porto, Portugal). Purified PCR products from each DGGE band were cloned using a pGEM®—T Easy Vector System Kit (Promega, Madison, WI, USA), and transformed into *Escherichia coli* ONE SHOT® TOP10 chemically competent cells (Invitrogen, San Diego, CA), following the instructions of the manufacturers. Colonies were selected by blue-white screening, and the presence of the appropriate insert was evaluated by colony PCR, using the primers pUCF/pUCR. Colonies with the insert were grown overnight at 37°C, in liquid LB medium supplemented with 100 μg ml^−1^ of ampicillin, with shaking at 200 rpm and plasmids were isolated from the overnight cultures using the GenElute Plasmid Miniprep Kit (Sigma, USA).

### Sanger sequencing

Purified plasmids and PCR products obtained from isolates (purified with the same spin columns mentioned above) were sent for sequencing at Macrogen (Amsterdam, Netherlands). All sequences were checked for chimera formation using the software DECIPHER (Wright et al., 2012).

### High-throughput amplicon sequencing

PCR amplifications from eDNA were obtained using the same primers used for PCR-DGGE, but without the GC clamp in the forward primer. They were originally designed (Nübel et al., 1997) to target the V3–V4 region of the 16S rRNA gene for cyanobacteria (including chloroplasts). This region is suitable for studying cyanobacterial diversity by NGS methodologies (Mizrahi-Man et al., 2013; Nguyen et al., 2016). The amplification of PCR products was carried out using a barcode-tagged PCR primer approach, following the same conditions, adaptors and reagents as described in Pinto et al. (2014). Pre-sequencing processing such as amplicon library generation, barcoding and emulsification are described elsewhere (Pinto et al., 2014). Massive parallel sequencing was performed using the Genome Sequencer FLX System Instrument (454 Life Sciences, Roche) at Biocant, Portugal. Raw sequence reads were then analyzed and processed using an in-house, automatic pipeline from Biocant, Portugal. Processing steps performed included sorting of sequences by sample, dereplication, filtering of low-quality sequences, detection and removing of DNA chimeras, Operational Taxonomic Units (OTUs) clustering (sequence similarity cutoff value of 97%), and generation of OTUs consensus sequences, as described in Pinto et al. (2014). Steps of quality control included the exclusion from further processing of putative contaminations and artifacts, of reads < 100 aligned nucleotides or with a low alignment quality, of reads with more than 2% of ambiguities, or 2% of homopolymers, and of singleton reads (i.e., a read with a sequence that is present exactly once).

In order to compare between OTU delineation methods, raw reads obtained were additionally analyzed in the SILVAngs pipeline (Quast et al., 2013). After removing primers and barcode tags, reads were dereplicated and unique reads with a sequence similarity value of 98% were clustered into OTUs. The same above mentioned steps of quality were followed. The reference read of each OTU (i.e., the longest read in each cluster) was classified by a local BLASTn search against the non-redundant version of the SILVA SSU Ref dataset (release 123; http://www.arb-silva.de) with standard settings (Camacho et al., 2009).

### Nucleotide sequence accession numbers

Novel PCR-based sequences associated with this study are available in GenBank under the accession numbers KT730170-KT730215. Sequence reads obtained in this study were deposited in NCBI's Sequence Read Archive (SRA) with the project number PRJNA294527 (SRA identifier: SRP063335); for corresponding accession numbers and further details on sequences see Supplementary Table S2.

### Phylogenetic analysis

The cyanobacterial 16S rRNA gene sequences from isolates (9), DGGE bands (38), and consensus sequences of pyrosequencing derived “97% cutoff” OTUs (105) were analyzed phylogenetically (Table 2 and Supplementary Table S2). OTUs consensus sequences with < 300 nucleotides length were removed from phylogenetic and downstream analyses. A second round of identification and removal of chimeras was performed for pyrosequencing sequences using DECIPHER (Wright et al., 2012). In order to include the most similar sequences and to attain a reliable and robust backbone representation of the cyanobacterial diversity, the best BLAST hits for our sequences (and the closest known relative, if the best hit was an unidentified organism) were also included in the phylogeny (see Supplementary Table S2), together with all the available sequences from reference strains included in the Bergey's Manual of Systematic Bacteriology (Castenholz et al., 2001). The sequences from the unidentified melainabacterium strain YS2 and *Chloroflexus auranticus* J-10-fl were used as outgroups.

**Table 2:** Number of cyanobacterial 16S rRNA gene sequences used in or discarded from phylogeny, by sample.

**Sequences from**	**EB1**	**EB2**	**EB3**	**Total**
**Included**				**(145)**
Isolates	4	3	2	9
DGGE bands	8	13	17	38
Pyrosequencing OTUs^#^	64	24	10	98
**Not included**				**(67)**
Singlets	27	18	9	54
Sequences <300 bp^§^	4	1	2	7
Chimeras^*^	0	2	4	6

# *Sequence similarity threshold of 97%; generated by the Biocant pipeline*.

§ *But included in further analyses (e.g., see Table 6)*.

* *Detected in a second round of screening, using DECIPHER*.

Multiple sequence alignment, evolutionary analyses and phylogenetic tree reconstructions were carried out using the software package MEGA6 (Tamura et al., 2013). Kimura 2-parameter was the model of nucleotide substitution used to infer the Maximum Likelihood (ML) tree (1,000 replicates), as chosen by the corrected Akaike's Information Criterion (AICc). A discrete Gamma distribution was used to model evolutionary rate differences among sites [5 categories (+G, parameter = 0.3556)]. The rate variation model allowed for some sites to be evolutionarily invariable ([+I], 31.8460% sites). The final analysis involved 402 nucleotide sequences with a total of 345 positions in the dataset.

### Taxonomic assignments and cyanobacterial diversity comparison

Cyanobacteria were identified based on morphology (hereafter referred to as morphospecies) following taxonomic identification keys from Komárek and Anagnostidis (1998, 2005). All taxa were then brought to their most recent taxonomic synonyms (Guiry and Guiry, 2016), following the recent system of classification at the genus and order level (Komárek et al., 2014). This list of morphospecies was compared with the list of morphospecies acquired from the survey (Supplementary Table S1), which were also brought to the most recent synonyms (Guiry and Guiry, 2016).

Cyanobacterial 16S rRNA gene sequences obtained in this study were classified by means of different automatic, hierarchical taxonomies such as Greengenes v13.8 (McDonald et al., 2012), RDP II classifier v11.4 (Wang et al., 2007), NCBI Taxonomy (Federhen, 2012), and SILVA Taxonomy v123 (Quast et al., 2013) using standard settings. Furthermore, using a phylogeny-guided clustering approach as recommended by Nguyen et al. (2016), we have manually curated and categorized the sequences into phylotypes according to their phylogenetic placement and bootstrap support of clades (Supplementary Image S3). Thus, in this study, a phylotype should be taken as a taxon *sensu lato*, which may embrace diversity corresponding to more than one traditional, taxonomic rank.

The number of taxa (“species” richness, S) determined by morphological- (i.e., morphospecies) and DNA-based approaches (i.e., phylotypes and OTUs defined by a 97% or a 98% identity threshold) were compared between samples and among methods. Furthermore, using the number of reads encompassed in each OTU (97%) or phylotype, i.e., their relative abundance (Supplementary Tables S2, S3), other diversity indices were calculated (according to Morris et al., 2014) and compared between samples: Shannon's diversity (*H'*), Simpson's diversity (*1/D*), and Shannon's evenness (*E*_*H*_).

## Results

### Characterization of microbial mats

Morphologically, the cyanobacterial mats found at the sampling sites belonged to the smooth (EB1) or polygonal (EB2 and EB3) types, while structurally they were layered (Supplementary Table S1 and Supplementary Image S1). At the time of sampling, smooth mats from EB1 presented a carpet-like form, covering a large area of the pond bottom. The sampled mat had a thin green layer on top (about 3.5 mm), followed by a purple (5.5 mm), and a dark (5.48 mm) layer (Supplementary Image S1G). The mats found in EB2 and EB3 consisted of large, irregularly shaped (due to border wear) polygonal plates (Supplementary Images S1B,C). Sampled mats (Supplementary Images S1H,I) showed a thin yellow-greenish layer on top (2.4 and 2.7 mm in EB2 and EB3, respectively), followed by a purple-brown layer (2.9 and 3.4 mm) and then a dark layer (24.6 and 27.9 mm). Some thin and discontinuous calcium carbonate layers were found below the cyanobacterial layer (data not shown).

### Morphological characterization of cyanobacterial diversity

**Figure 3:**
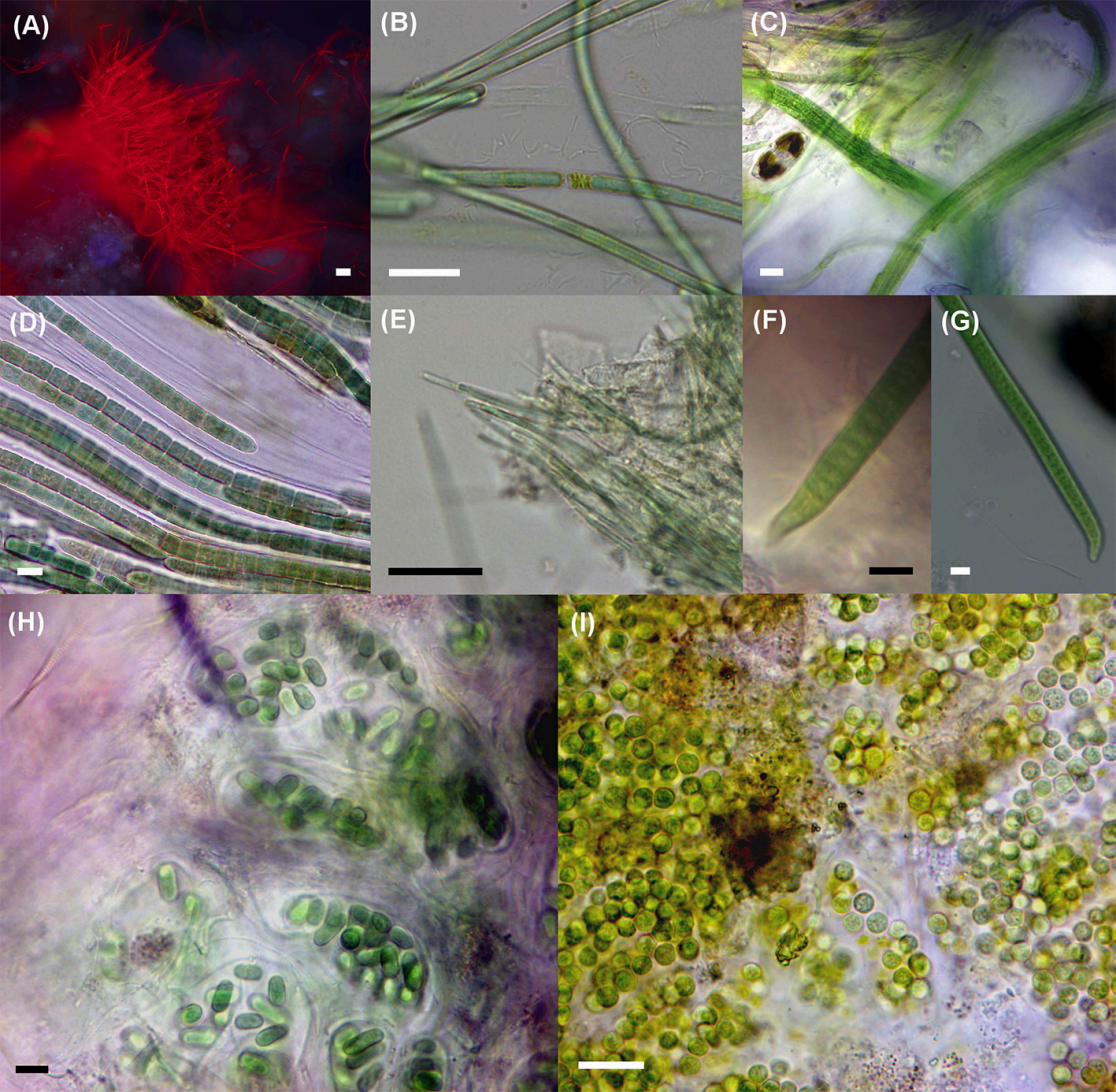
Epifluorescence **(A)** and bright field micrographs **(B–I)** showing ubiquitous, abundant, or dominant cyanobacteria in the environmental samples. **(A)** Tuft of filaments from *Halomicronema excentricum*, a thin cyanobacterium common to the three samples and abundant in the mat from EB2; **(B)** *Geitlerinema* cf. *lemmermannii*, present in the three samples and being dominant at EB1 and abundant at EB3; **(C)** *Microcoleus* aff. *steenstrupii*, abundant in EB1; **(D)** *Coleofasciculus chthonoplastes*, a dominant species in mats collected at EB2 and EB3; **(E)** *Halomicronema excentricum*; **(F,G)** *Oxynema* cf. *lloydianum* abundant at EB3; **(H)** *Aphanothece* cf. *stagnina* and **(I)** *Geminocystis* sp. both common to all three samples. Scale bar: 10 μm.

Thirty-six morphospecies belonging to 22 genera were distinguished by microscopic observations of the three mat samples (Table 3, Figure 3, and Supplementary Images S4–S6). In the mat collected at EB1 we observed 21 species, 18 in EB2 and 12 in EB3, belonging to the orders Chroococcales, Oscillatoriales, Spirulinales, and Synechococcales. Members of the Nostocales, Pleurocapsales, or Chroococcidiopsidales were not observed in any of the samples. The most represented genera were *Aphanothece, Oscillatoria, Spirulina* (only from the EB3 sample), and *Pseudanabaena*.

**Table 3:** Species composition and morphological-based characterization of cyanobacteria observed in the upper layer of the microbial mats collected at Araruama (EB1), Pitanguinha (EB2), and Pernambuco (EB3) lagoons.

**Taxa (Order/Morphospecies)**	**Sample**^a^			**Cell size^b^(Ø or length × width; in μm)**	**Figures/panels**
	**EB1**	**EB2**	**EB3**		
**CHROOCOCCALES**					
*Aphanothece* cf. *conglomerata* Rich	•			1.3 ± 0.1 × 0.9 ± 0.1	S4C
*Aphanothece* aff. *salina* Elenkin & Danilov	•			6.0 ± 1.2 × 3.5 ± 1.0	S4D
*Aphanothece* cf. *stagnina* (Sprengel) A.Braum	•	°	°	9.5 ± 1.9 × 4.6 ± 0.6	3H and S4E
*Chroococcus* aff. *turgidus* (Kützing) Nägeli	•			22.9 ± 3.2 × 33.2 ± 5.1	S4F
*Cyanosarcina* aff. *thalassia* Anagnostidis & Pantazidou		°		1.3 ± 0.2	S4H
*Geminocystis* sp. Korelusová, Kastovský & Komárek	•	•	•	4.1 ± 0.8	3I and S4I
*Gloeocapsopsis* cf. *crepidinum* (Thuret) Geitler ex Komárek	•	°		4.3 ± 1.3	S4J
*Gloeothece* cf. *subtilis* Skuja	•			1.5 ± 0.1 × 0.8 ± 0.1	S4K
**OSCILLATORIALES**					
*Coleofasciculus chthonoplastes* (Thuret ex Gomont) Siegesmund, Johansen & Friedl		□	□	8.0 ± 2.0 × 6.5 ± 0.7	3D and S5A
*Geitlerinema* aff. *amphibium* (Agardh ex Gomont) Anagnostidis		•		5.2 ± 0.7 × 2.8 ± 0.5	S5B
*Geitlerinema* cf. *lemmermannii* (Woloszynska) Anagnostidis	■	•	■	5.2 ± 1.3 × 2.1 ± 0.3	3B and S5C
*Microcoleus* aff. *steenstrupii* Petersen	■	•		6.3 ± 1.3 × 3.9 ± 0.6	3C and S5I
*Oscillatoria limosa* Agardh ex Gomont		•	°	4.4 ± 0.7 × 9.7 ± 0.3	S5L
*Oscillatoria margaritifera* Kützing ex Gomont		•		6.2 ± 0.7 × 27.6 ± 1.9	S5M
*Oscillatoria subbrevis* Schmidle		•		2.5 ± 0.4 × 7.3 ± 0.3	S5N
*Oxynema* cf. *lloydianum* (Gomont) Chatchawan, Komárek, Strunecky, Smarda & Peerapornpisal		•	■	3.4 ± 0.7 × 8.5 ± 0.8	3F–G and S5O
*Phormidium nigroviride* (Thwaites ex Gomont) Anagnostidis & Komárek		•		4.5 ± 0.8 × 14.3 ± 0.7	S5P
**SPIRULINALES**					
*Spirulina labyrinthiformis* Gomont			•	0.7 ± 0.1 wide, spirals width: 1.9 ± 0.5	S6B,C
*Spirulina subsalsa* Oerstedt ex Gomont			°	1.3 ± 0.3 wide, spirals width: 3.4 ± 0.3	S6D
*Spirulina tenerrima* Kützing ex Gomont			•	0.4 ± 0.1 wide, spirals width: 1.4 ± 0.1	S6E
**SYNECHOCOCCALES**					
*Aphanocapsa litoralis* (Hansgirg) Komárek & Anagnostidis	•			3.0 ± 0.5	S4A
*Aphanocapsa* cf. *salina* Woronichin	•	•		1.2 ± 0.2	S4B
*Coelosphaeriopsis* cf. *halophila* Lemmermann	•			2.8 ± 0.2	S4G
*Halomicronema excentricum* Abed, Garcia-Pichel & Hernández-Mariné	•	■	•	2.8 ± 0.6 × 0.8 ± 0.1	3E and S5H
*Komvophoron breve* (Carter) Anagnostidis	•			1.1 ± 0.1 × 2.0 ± 0.2	S5D
*Komvophoron* cf. *minutum* (Skuja) Anagnostidis & Komárek			•	2.0 ± 0.3 × 2.2 ± 0.3	S5E
*Lemmermanniella* sp. Geitler in Engler & Prantl	•			1.0 ± 0.1 × 0.6 ± 0.1	S4L
*Leptolyngbya crosbyana* (Tilden) Anagnostidis & Komárek	•	•		1.9 ± 0.6 × 1.3 ± 0.1	S5F
*Leptolyngbya* cf. *ectocarpi* (Gomont) Anagnostidis & Komárek	•			3.0 ± 1.0 × 1.5 ± 0.4	S5G
*Nodosilinea* sp. Perkerson & Casamatta		•		1.7 ± 0.5 × 2.5 ± 0.5	S5J
*Nodosilinea nodulosa* (Li & Brand) Perkerson & Casamatta	•			1.0 ± 0.2 × 1.1 ± 0.2	S5K
*Pseudanabaena* aff. *amphigranulata* (Goor) Anagnostidis		•		3.8 ± 0.9 × 1.3 ± 0.1	S5Q
*Pseudanabaena* cf. *limnetica* (Lemmermann) Komárek	•			3.5 ± 1.1 × 0.8 ± 0.3	S5R
*Pseudanabaena* cf. *minima* (G.S.An) Anagnostidis	•			1.5 ± 0.5 × 1.4 ± 0.2	S6A
*Synechococcus* sp. Nägeli			•	2.1 ± 0.6 × 0.8 ± 0.1	S4M
*Synechocystis salina* Wislouch	•			2.1 ± 0.2	S4N

a *Underlined symbols refer to first reported observations for the entire lagoons complex. Black circles or squares denote first reported observations of a species for that water body. White circles or squares indicate that the species was also observed in previous studies (see also the full checklist available in Supplementary Table S1). Squares mean that the taxon was found to dominate or was abundant in samples from this study*.

b *Mean ± standard deviation values (n = 20)*.

Four morphospecies were common to all three samples (Table 3): the colonial, rod-shaped *Aphanothece* cf. *stagnina* (Figure 3H), the spherical, unicellular *Geminocystis* sp. (Figure 3I), the very thin, filamentous *Halomicronema excentricum* (Figures 3A,E), and the highly motile, filamentous *Geitlerinema* cf. *lemmermannii* (Figure 3B). This latter species dominated the mat sample from EB1 and was abundant at EB3. *Microcoleus* aff. *steenstrupii* (Figure 3C), with trichomes densely packed in fascicles, was abundant at EB1, and present at EB2. The wide sheathed, bundle-forming species *C. chthonoplastes* (Figure 3D) dominated the mats collected at EB2 and EB3, but was not observed in EB1. Other abundant taxa were *Leptolyngbya minuta* (EB2) and *Oxynema* cf. *lloydianum* (Figures 3F,G) at EB3. *O. lloydianum* was also detected at EB2. Although, visibly dominated by cyanobacteria, the microscopic examination indicated the presence of other organisms in the top layer of the mats (Supplementary Image S6).

Nine cyanobacterial strains belonging to five different taxa were isolated (Table 4 and Figure 4). One taxon is from the order Oscillatoriales (*Geitlerinema* cf. *lemmermannii*) and the other four from the order Synechococcales (*Leptolyngbya* aff. *ectocarpi, Leptolyngbya* sp., *Nodosilinea* sp., and *Synechococcus* sp.).

**Table 4:** List of cyanobacterial strains isolated from hypersaline microbial mats collected at the Araruama lagoon system (RJ, Brazil).

**Strain**	**Order**	**Sampling site**	**Figure**
*Leptolyngbya* aff. *ectocarpi* LEGE 11389	Synechococcales	EB1	4B
*Geitlerinema* cf. *lemmermannii* LEGE 11390	Oscillatoriales	EB1	4E
*Geitlerinema* cf. *lemmermannii* LEGE 11391	Oscillatoriales	EB1	4G
*Leptolyngbya* sp. LEGE 11392	Synechococcales	EB3	4D
*Geitlerinema* cf. *lemmermannii* LEGE 11393	Oscillatoriales	EB2	4F
*Synechococcus* sp. LEGE 11394	Synechococcales	EB3	4A
*Nodosilinea* sp. LEGE 11395	Synechococcales	EB2	4C
*Geitlerinema* cf. *lemmermannii* LEGE 11396	Oscillatoriales	EB1	4H
*Geitlerinema* cf. *lemmermannii* LEGE 11401	Oscillatoriales	EB2	^#^

# *This strain was lost*.

**Figure 4:**
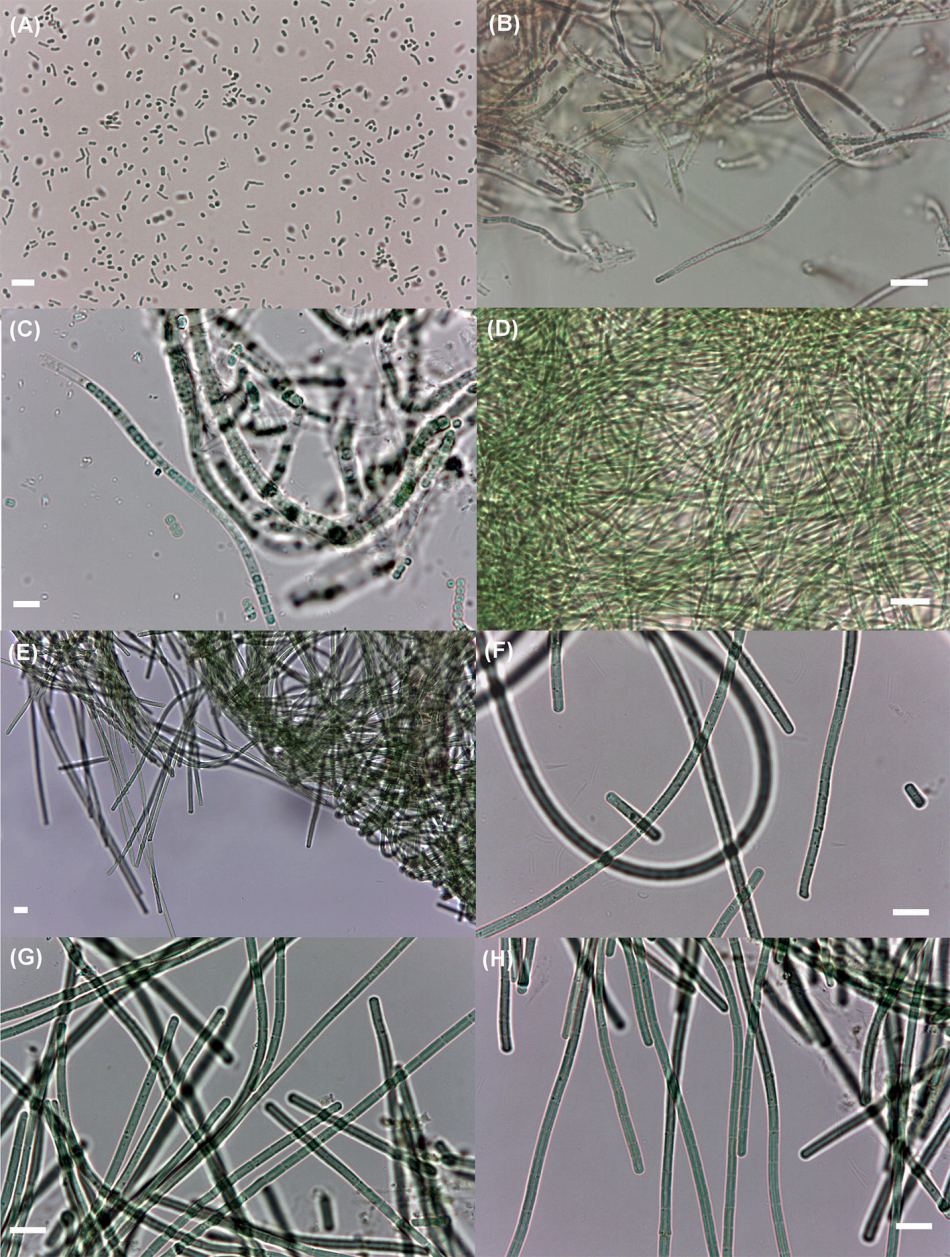
Cyanobacterial isolates obtained in this study. **(A)** The small, unicellular *Synechococcus* sp. LEGE 11394; **(B)** the brownish, filamentous *Leptolyngbya* aff. *ectocarpi* LEGE 11389; **(C)** the sheathed filamentous *Nodosilinea* sp. LEGE 11395; **(D)** the thin, filamentous *Leptolyngbya* sp. LEGE 11392; **(E)** *Geitlerinema* cf. *lemmermannii* LEGE 11390, at 400× magnification; **(F–H)** the same non-sheathed filamentous species as in **(E)**, at 1,000× magnification; strains LEGE 11393, 11391, and LEGE 11396, respectively. The short filaments in **(F)** are hormogonia. Scale bar: 10 μm.

### Molecular and phylogenetic characterization

**Figure 5:**
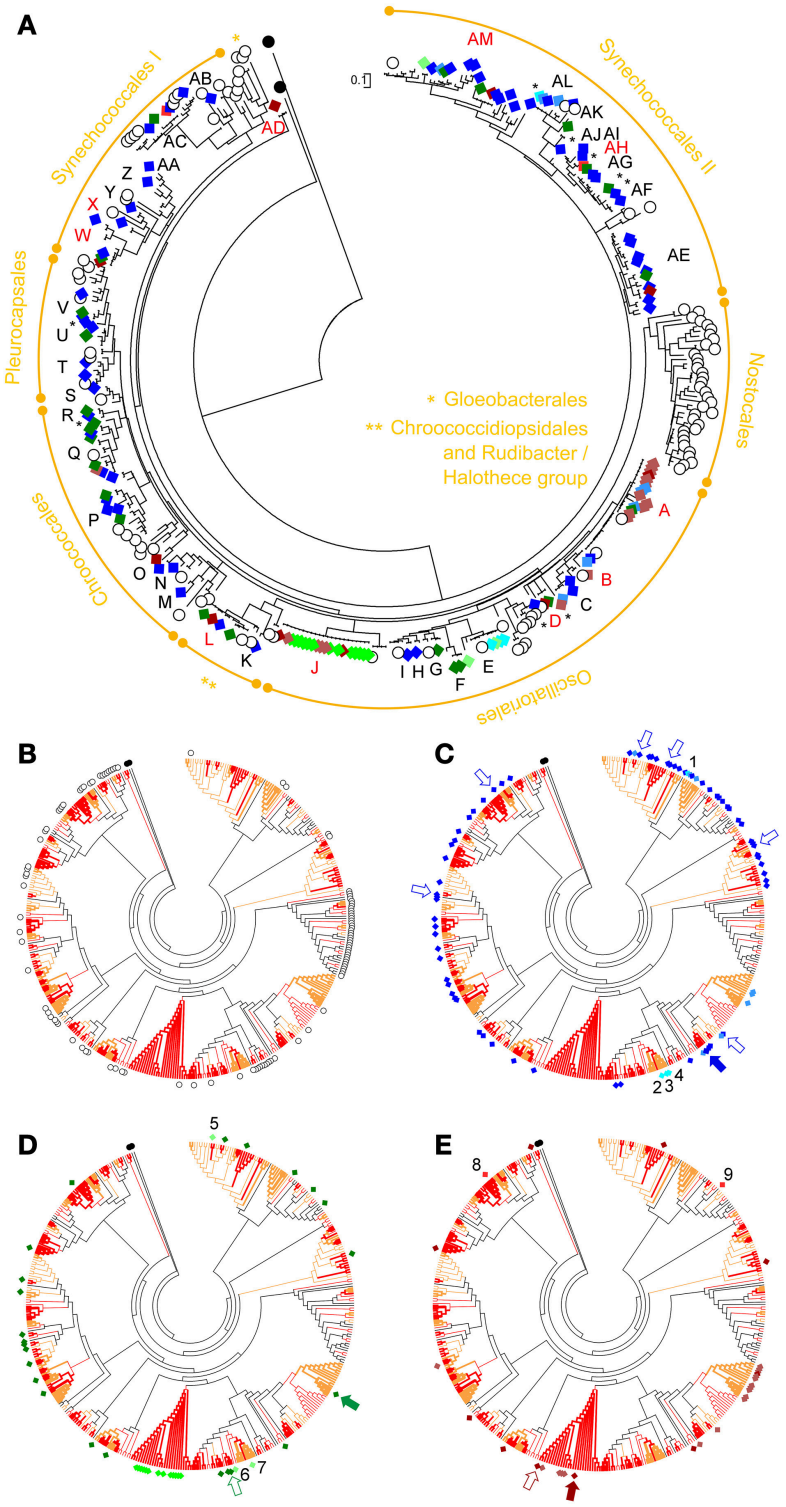
Circular phylogenetic ML trees (log-likelihood of −15279.7163) based on cyanobacterial 16S rRNA gene sequences. Sequences obtained in this study are marked with colored diamond squares (see below), while white circles denote sequences from reference strains. The phylogram tree in **(A)** shows the taxonomic classification for the sequences, at the order level, and their assigned phylotypes (capital letters); highlighted in red are phylotypes that also include sequences obtained from hypersaline microbial mats from Guerrero Negro (see Harris et al., 2013 and text for details). Black asterisks indicate phylotypes consisting in single sequences that have no close relatives (i.e., loner sequences *sensu* Wilmotte and Herdman, 2001). The trees in **(B–E)** are the cladogram version of the tree in **(A)**; tree branches in orange represent values of bootstrap support >50%, and in red >75% (1,000 replicates). In **(B)** are highlighted the reference strains sequences; in **(C)** the sequences from the mat collected at EB1 (Araruama lagoon); in **(D)** those from EB2 (Pitanguinha); and in **(E)** the sequences from EB3 (Pernambuco). Bluish diamonds indicate sequences from EB1, greenish are from EB2, and brownish are from EB3. Darker colors refer to 454-OTUs, lighter to isolates and normal colors are for DGGE-derived sequences. Numbers in **(C–E)** highlight the isolates obtained from each mat: 1 *Leptolyngbya* aff. *ectocarpi* LEGE 11389; 2, 3, and 4 *Geitlerinema* cf. *lemmermannii* strains LEGE 11390, 11391, and 11396; 5 *Nodosilinea* sp. LEGE 11395; 6 and 7 *Geitlerinema* cf. *lemmermannii* strains LEGE 11393 and 11401; 8 *Synechococcus* sp. LEGE 11394; 9 *Leptolyngbya* sp. LEGE 11392. Arrows point out all OTUs encompassing more than 4% of the total pyrosequencing reads from a sample. In addition, filled arrows indicate the most abundant OTU of each sample. Tree was rooted with the unidentified melainabacterium strain YS2 (AF544207) and *Chloroflexus aurantiacus* J-10-f (CP000909) as outgroups.

The pyrosequencing generated 10,836 high quality reads in total, for the three mat samples. The number of reads was decreased to 10,487 after removing singletons (54 were from cyanobacteria; see Table 2). The number of OTUs obtained showed a similar pattern for the three samples in rarefaction curves with a good coverage (Supplementary Image S8). Cyanobacterial 16S rRNA gene sequences accounted for >85% of total reads in any of the samples, while plastid sequences only accounted for ≤ 0.8% in any of the mats (see also Supplementary Images S7, S9). A circular ML tree (Figure 5) with 145 16S rRNA gene sequences obtained in this study (Table 2) was generated, along with sequences from reference strains (Castenholz et al., 2001) and from BLAST search results. Additional visualizations of the same tree are provided in Supplementary Images S3, S10. The sequences obtained in this study are distributed across the entire tree with the exception of the Nostocales and Gloeobacterales clades (Figure 5A). The same holds true when looking at sequences from each mat (Figures 5C–E). For the EB1 sample (Figure 5C), the most abundant 97% OTU, (20.6% relative abundance) is placed in the clade of phylotype C in the Oscillatoriales (see also Supplementary Image S3). Six other OTU sequences, placed in phylotypes from different lineages of Synechococcales, Pleurocapsales, or Oscillatoriales, had over 4% relative abundance. These observations contrast with the pyrosequencing data for mats collected at the other two sites. The mat from EB2 (Figure 5D) was clearly dominated by a single OTU (84.7% relative abundance). This sequence is placed in phylotype A, which includes the reference strains *Coleofasciculus* (ex-*Microcoleus*) *chthonoplastes* CCY9606 and PCC 7420 (Siegesmund et al., 2008; Supplementary Image S3). The mat from EB3 (Figure 5E) was also dominated by a single sequence (85.9% relative abundance). It was included in phylotype J, which also encompasses the second most abundant sequence in the sample (>4% relative abundance) (Supplementary Image S3).

Regarding the sequences from excised DGGE bands, those from the EB1 and EB3 mats were placed among different lineages of the tree (see also Supplementary Image S3 and Supplementary Table S2). The DGGE band sequences from EB2 were all placed in the clade of phylotype J.

The isolate-derived sequences were found to belong to different lineages of the order Synechococcales or to the same lineage within the Oscillatoriales (phylotype E), as shown Figure 5. The clade of this latter phylotype contains the reference strain *Geitlerinema* sp. PCC 7105. These findings are in accordance with the morphological-based identification (Table 4).

Seventy-five sequences (51.7% of total) obtained in this study had a best BLAST hit result of ≥99% (Supplementary Table S2). From these, 51 sequences had as best hit a sequence from a saline environment. The majority of these homologous sequences (42 out of 51) were obtained from hypersaline microbial mats collected at a single location—Guerrero Negro, Baja California Sur, Mexico (Harris et al., 2013). The Araruama's and the highly similar Guerrero Negro's hypersaline cyanobacterial sequences grouped into nine distinct lineages (phylotypes A, B, D, J, L, W, X, AD, AH, and AM; Figure 5; see also Supplementary Table S2 and Supplementary Image S3).

The metagenomic data obtained using the SILVAngs pipeline, (98% OTUs cutoff) can be visualized as Krona charts (Ondov et al., 2011) in a permalink that was archived by WebCite at http://www.webcitation.org/6kiUALfVA (see also Supplementary Image S9).

### Richness, diversity, and species composition comparisons

**Figure 6:**
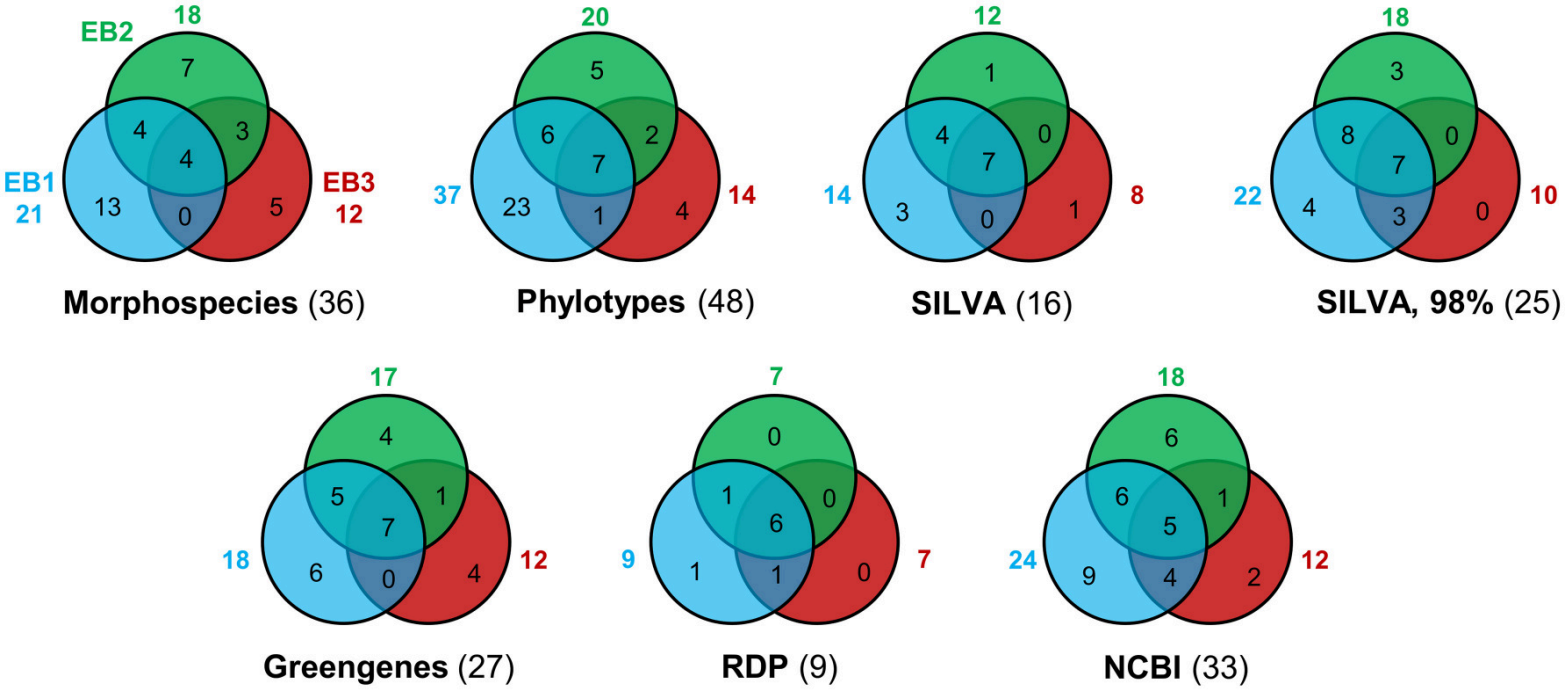
Venn diagrams showing the number of distinct cyanobacterial taxa distinguished in each mat sample, by different approaches (including a morphological-based identification, a phylogenetic-guided categorization or an automatic taxonomic classification using different classifiers). If not explicitly specified, OTU consensus sequences were defined as a cluster of reads with 97% similarity. In parentheses are the number of unique taxa identified in all samples.

Taxon richness (*S*) values obtained by the different approaches is illustrated by Venn diagrams (Figure 6). Regardless of the method used, EB1 was invariably the mat that showed a higher number of taxa (Figure 6). In contrast, EB3 was the mat with the lowest number of taxa (the only exception was with the RDP classifier, for which EB3 had same taxon richness as EB2). The number of common taxa present in all three mats varied from four (morphological-based identification) to seven. There were more taxa shared by EB1 and EB2 than by EB1 and EB3, or by EB2 and EB3. With regard to classification methods, the number of unique taxa recognized in all samples was higher when looking at phylotypes (48 taxa), morphospecies (36) or at sequences classified using the NCBI Taxonomy database (33). The RDP classifier had the lowest performance in differentiating the cyanobacterial diversity (9) present on the mats from Araruama's lagoons. With the exception of the NCBI Taxonomy database, a considerable number of unclassified sequences was obtained by the classifiers (see Supplementary Table S2). The most stringent definition of OTU (98%) increased the number of distinct taxa obtained (25 vs. 16 for the 97% threshold).

Table 5 shows the species richness by cyanobacterial order and compares values obtained in this study for morhospecies and phylotypes, with the number of morphospecies previously reported for the Araruama lagoon system, as recovered from our survey (Supplementary Table S1). With 21 new cyanobacterial species records and eight new genera records, this study has increased by 16.3 and 20% the number of (morpho-) species and genera reported for the Araruama system, respectively (Table 3). Similar results were obtained for taxonomic assignments of morphospecies and phylotypes at the order level (Table 5). The main differences were the identification of Spirulinales species by the morphological-based approach, an order not detected in the 16S rRNA-based phylogeny of Araruama's sequences, and the detection of phylotypes within the Pleurocapsales, Chroococcidiopsidales, and *Halothece*-related lineages, taxa that we were unable to identify by microscopic examination.

**Table 5:** Taxon richness comparison, by taxonomic order, of morphospecies and phylotypes identified in this study and morphospecies previously reported for lagoons from the Araruama's entire complex, as retrieved from the literature survey (see Supplementary Table S1 for the full checklist).

**Order**	**Number of morphospecies (this study)^#^**	**Number of unique phylotypes (this study)**	**Number of morphospecies (other studies)^#^**	**Total number of morphospecies, including this study^#^**
Chroococcales	8 (6), 1 new record^&^	8 [including 1 loner sequence]	46 (14)	47 (15)
Chroococcidiopsidales	0	1	1 (1)	1 (1)
Gloeobacterales	0	0	0	0
Nostocales	0	0	4 (3)	4 (3)
Oscillatoriales	9 (6), 6 new	12 [2 loner sequences]	27 (9)	33 (10)
Pleurocapsales	0	4 [1 loner sequence]	1 (1)	1 (1)
*Rudibacter*/*Halothece* group^*^	0	1	0	0
Spirulinales	3 (1), 2 new	0	5 (1)	7 (1)
Synechococcales	16 (10), 12 new	22 [5 loner sequences]	24 (11)	36 (17)
Total	36 (22), 21 new records	48 [9 loner sequences]	108 (40)	129 (48)

# *Number of genera shown in parentheses*.

& *New species records for Araruama's entire system*.

* *This is a cyanobacterial lineage that will probably give origin to a separate order, as stated in Komárek et al. (2014). For now, these genera are placed within the order Chroococcales (Komárek et al., 2014). Some OTUs belonging to this lineage were previously detected by Clementino et al. (2008), in water samples from Araruama's main lagoon. Moreover, the genus Halothece may actually contain some species of Aphanothece (order Synechococcales), including A. halophytica, a halophilic species common in hypersaline environments (see Oren, 2012 for taxonomic details)*.

The cyanobacterial species richness estimates for the samples, obtained after applying morphological- or phylogenetically-based, manually curated classifications, or just after clustering of OTUs directly derived from metagenomic data are depicted in Table 6. This table also shows other diversity measures for the cyanobacteria present in the mat samples for a 97% OTU cutoff. The value of *S* was higher for unclassified OTUs than for morphospecies or phylotypes, and was also higher for the more stringent 98% OTU cutoff. Irrespectively of the type of taxa categorization, *S* was consistently higher for the EB1 and lower for the EB3 mat samples. In general, *H'* and *1/D* values were consistent with these observations among samples and between the two types of taxa categorization. *E*_*H*_ estimates were also higher for the EB1 sample and lower for EB3 (phylotypes) or for EB2 (97%-level OTUs).

**Table 6:** Diversity estimates, considering different categorizations of taxa and/or molecular data processing.

**Mat sample**	**“Species” richness (*****S*****)**				**Shannon's diversity (*****H'*****)**		**Simpson's diversity (*****1/D*****)**		**Shannon's evenness (*****E_H_*****)**	
	**Morphospecies^&^**	**Phylotypes^&^**	**97% OTUs^#^**	**98% OTUs^#^**	**Phylotypes^$^**	**97% OTU^$^**	**Phylotypes^$^**	**97% OTU^$^**	**Phylotypes^$^**	**97% OTU^$^**
EB1	21	37	68	258	2.57	3.12	8.87	12.41	0.71	0.74
EB2	18	20	25	128	0.65	0.72	1.39	1.39	0.22	0.22
EB3	12	14	12	68	0.42	0.59	1.23	1.35	0.16	0.24

& *See also Figure 6*.

# *Unclassified pyrosequencing OTUs; directly derived from Biocant or SILVAngs metagenomic pipelines according to a 97 or 98% identity threshold for clustering, respectively*.

$ *Abundances from 454 pyrosequencing-derived data (see Supplementary Table S2); based on the total number of reads that gave rise to each OTU (i.e., consensus) sequence*.

## Discussion

In this work, we have attempted to uncover the cyanobacterial diversity present in hypersaline mats from three lagoons of the Araruama system, while exploring the impact of different classification methods or procedures to evaluate such diversity. The polyphasic approach used confirmed and extended the high cyanobacterial diversity reported previously in morphological-based studies, for the entire Araruama system (see Supplementary Table S1). The differences in terms of diversity observed between EB2/EB3 and EB1 (Table 6 and Figure 6) may have been caused by the lower salinity observed for EB1 when compared to the other two sites. However, it is also possible that a bigger sampling effort (i.e., larger sampled area) could have resulted in more taxa overlap among the studied mats.

### Molecular-based approach

As expected, pyrosequencing allowed a deeper coverage of the diversity present in the samples, particularly when compared to PCR-DGGE (Table 2). In fact, PCR-DGGE revealed less diversity than we had anticipated—which might be partially explained by our inability to excise many of the abundant faint bands that were observed (Supplementary Image S2; see also Sánchez et al., 2009). Amplification bias is also a known issue in PCR-DGGE (Neilson et al., 2013) and may explain underrepresentation of certain taxa in our data. Curiously, the DGGE-detected phylotypes J (EB2 sample) and A (EB1 sample) could not be detected by pyrosequencing (Figure 5) despite the same pool of DNA having been used for both techniques (Figure 2). Like other PCR-based approaches (von Wintzingerode et al., 1997; Speksnijder et al., 2001), the two culture-independent molecular techniques employed in this study are prone to bias, artifacts, pitfalls, and have limitations, whose discussion and explanation falls beyond the scope of this study (for details on these issues see Mühling et al., 2008; Green et al., 2010; Berry et al., 2011; Scholz et al., 2012; Bragg and Tyson, 2014). It is possible that by removing singletons and small sequences from the pyrosequencing data (Table 2), we may have missed sequences phylogenetically close to the unique DGGE-derived sequences. Still, because the DGGE and pyrosequencing datasets were not entirely redundant, using both techniques proved a fruitful strategy.

### Culture-dependent approach

Five of the nine isolates corresponded to a single species, *Geitlerinema* cf. *lemmermannii* (Table 4 and Figure 5). *Geitlerinema* spp. are common in hypersaline microbial mats (Richert et al., 2006; Goh et al., 2009). Hence, obtaining isolates from this genus facilitates future studies on the ecophysiology of these organisms in hypersaline mats. Quite surprisingly, the clade of this phylotype does not include any sequence obtained by the culture-independent approaches (Supplementary Image S3), even though this same morphospecies was detected by microscopy in all samples and shown to be abundant in EB1 and EB3 (Table 3 and Figure 3B). One possible explanation is bias in the DNA extraction from this cyanobacterium from environmental samples (gDNA extraction from cultured isolates was not problematic). Nevertheless, this finding reinforces the relevance of using complementary methodologies. The usefulness of culturing due to its capacity to unveil novel microbial diversity, undetected by current metagenomics techniques is well-known (Lagier et al., 2015). In line with these observations, the phylogenetic position of *Leptolyngbya* sp. LEGE 11392 (Figure 5 and Supplementary Image S3) indicates that its 16S rRNA gene qualifies as a loner sequence (*sensu* Wilmotte and Herdman, 2001). Hence, this isolate represents in all likelihood a cryptic taxon within the recognized polyphyletic genus *Leptolyngbya* (Komárek, 2016). This strain, very likely underrepresented in the original sample, will have been highly competitive during the isolation process.

The difficulty in bringing the observed diversity into culture, as portrayed by both the low number and low diversity of the isolates, suggests that improvements will have to be made in isolation strategies (e.g., circumscribe rapidly growing and mobile species, such as *Geitlerinema* cf. *lemmermannii*, by phototaxis), and cultivation (e.g., change culture media and/or make adjustments to their compositions to better mimic nutritional requirements of the cyanobacteria from hypersaline mats, or use culture medium specifically developed for some species, e.g., as for *Aphanothece halophytica* in Yopp et al., 1978). In fact, the most similar GenBank sequences (≥99%) for the sequences that we obtained were predominantly from saline environments (91%), evidencing a likely ecological specificity (e.g., salts or other nutrients) of the cyanobacteria living in this ecosystems, an issue that deserves further investigation.

### Comparison between molecular- and morphological-based approaches

For the first time, a molecular study was performed in order to characterize and classify the cyanobacterial diversity present in microbial mats from the Araruama's lagoons. The only available molecular sequences for cyanobacteria from this lagoon complex were from water samples, obtained from 16S rRNA gene and *nif* H clone libraries targeting the whole prokaryotic diversity (Clementino et al., 2008; see Supplementary Image S11 for a comparative phylogenetic tree). The 16S rRNA gene cyanobacterial sequences in Clementino et al. (2008) were phylogenetically placed within three different clades, one including *C. chthonoplastes* PCC 7420 (X70770), other with *Halothece* sp. PCC 7418 (AJ000708), and the third containing *Synechococcus* sp. WH8101 (AF001480), which, in turn, are included in the clades of phylotypes A, L, and AC, respectively, in our study (Figure 5 and Supplementary Image S3). The strains PCC 7420 and PCC 7418 belong to well-known halophilic or extremely halotolerant species (Garcia-Pichel et al., 1996; Oren, 2012). PCC 7420 was previously known as *Microcoleus chthonoplastes* but the taxonomy of this species was later revised to *C. chthonoplastes* (Siegesmund et al., 2008; see also nomenclatural comments in Oren, 2012). *Halothece* sp. PCC 7418 was firstly identified as *A. halophytica*, and is also known as *Cyanothece* sp. (Garcia-Pichel et al., 1998) due to confusing nomenclatural issues regarding related forms of *A. halophytica*, and which are better explained in Oren (2012). Clades of phylotypes A and L also harbor Araruama's closely-related sequences (>99% similarity) from Guerrero Negro (Harris et al., 2013; Figure 5). This location in Baja California, Mexico, contains one of the most well-studied hypersaline microbial mats, dominated by *C. chthonoplastes* (Garcia-Pichel et al., 1996; Stal, 2012). The close identity between the 16S rRNA gene sequences from several Araruama phylotypes and Guerrero Negro sequences suggests that these cyanobacterial lineages are ubiquitous in hypersaline environments.

Some congruence between phylogenetic placement and morphology-based identification could be observed. For instance, *C. chthonoplastes* was observed to dominate the samples from EB2 and EB3, but was not observed in EB1 (Table 3). Three *Aphanothece* spp. were detected, although none could be assigned to *A. halophytica*. The only *Aphanothece* species observed to be present in the three mats was *A*. cf. *stagnina* (Table 3), which could correspond to sequences in phylotype L (clade of *Halothece*), also observed in all studied mats. The picocyanobacterium *Synechococcus* sp. was detected by microscopy only in EB3 (Table 3), from the same sample from which *Synechococcus* sp. LEGE 11394 was successfully isolated (Table 4). Other good examples where the findings from both phylogeny and microscopy were similar include phylotype E, a clade with the reference strain *Geitlerinema* sp. PCC 7105 and all our *Geitlerinema* isolates (Figure 5, Table 4, and Supplementary Image S3), and phylotype AE, a clade with the Type strain *H. excentricum* TFEP1 (Figure 5 and Supplementary Images S3, S11), a very thin, filamentous Synechococcales that was also detected in all three samples by morphology and shown to be abundant in EB2 (Table 4 and Figure 3). The species *H. excentricum* was firstly described from microbial mats, in man-made solar ponds at Eilat, Israel (Abed et al., 2002). The clade of phylotype J, which is represented in EB2 and EB3 and very abundant in the latter (Figure 5 and Supplementary Table S3) contains a sub-clade with *Oscillatoria acuminata* PCC 6304 that very likely includes *Oxynema lloydianum* CCALA 960 (Chatchawan et al., 2012) (see Supplementary Images S3, S11). *Oxynema* cf. *lloydianum* is a morphospecies abundant in EB3 (Table 3), and characterized by having cylindrical filaments, narrowed and bent at their ends (Figures 3F,G), as described in Chatchawan et al. (2012). Despite these examples, most often a correspondence between morphospecies and phylotypes was not straightforward at the species/genus level (see, respectively, Table 3 and Supplementary Image S3). Still, at the order level, there was a good taxonomic correlation between the morphological identification and phylogeny, with several of the phylotypes being associated with the observed morphospecies (Table 5, see also Table 3 and Figure 5).

### Classification and identification issues

The assignment of taxa to sequences is often a challenge in molecular-based classification methods, chiefly in the analysis of metagenomic data directly retrieved from environmental samples (Mobberley et al., 2012; Garcia-Etxebarria et al., 2014; Tuzhikov et al., 2014). In order to define units of diversity, microbial ecologists very often rely on clustering of 16S rRNA sequences into OTUs. However, there is not a single satisfactory definition, and it is therefore common to observe the use of more flexible (>97% sequence identity) or more stringent (>98–99%) OTU delineations (Youngblut et al., 2013; Schmidt et al., 2015). Of course, this issue may have an impact on estimating species richness. This was the case for our dataset with a much higher diversity estimate when using the 98% OTU cutoff (Table 6).

In addition, cyanobacterial taxonomy is currently under revision and deals with several problematic issues (in particular, the recognition of the existence of cryptic species; Dvorák et al., 2015; Komárek, 2016) making it difficult to obtain a reliable identification at the species or genus level for some of these organisms. For instance, one of the reasons why the number of taxa generated by the automatic classifiers was smaller than that obtained by the two manually curated, i.e., morphospecies and phylotypes, classifications (Figure 6) is linked to the size of the classifier databases, namely an underrepresentation of the cyanobacteria phylum. Thus, due to the absence of proper reference sequences covering the cyanobacterial diversity, these databases may be unable to classify a significant part of a given data set (Garcia-Etxebarria et al., 2014; Tuzhikov et al., 2014) and lead to an underestimation of the number of unique taxa by comparison with other classification methods (Figure 6). Naturally, our manual curation process for classifying phylotypes, although quite laborious, was possible because a relatively small number of 454-read sequences were obtained (Table 2). The classification was based on a simple criterion, the bootstrap support of clades (Supplementary Image S3). This is still a broad, inexact demarcation of “taxa,” since clades may include lineages more or less divergent (i.e., sequences more or less similar), but ensures that phylogenetically close related sequences are grouped together.

In conclusion, it was shown that the three hypersaline mats studied harbor a high cyanobacterial diversity. Our morphological-based results increase by more than 15% the number of morphospecies and genera reported for all the lagoons of the Araruama coastal system. This fact is of particular relevance because an exhaustive examination of single samples, collected at each mat, was followed instead of studying diverse samples from each mat. The taxonomic/classification assignment methods and the different approaches used (namely culture-dependent and -independent methods) varied substantially in their ability to capture the diversity present in the samples. In our understanding, such approaches need to be regarded as complementary, and together enable a better understanding of cyanobacterial diversity in complex environmental samples. The phylogeny-guided sequence classification generated the highest number of unique taxa, although several could not be identified, at least at the genus level. In fact, only with the morphological-based approach was it possible to identify most of the recognized cyanobacteria present in the mat samples at lower taxonomic levels. At the order level, however, the taxonomic inferences were generally congruent between phylogeny and morphology.

## Author contributions

Conceived and designed experiments: VR, RC, PL, JM, VV. Performed the experiments: VR, RC, PL, JM, SC, FS. Wrote the paper: VR, RC, PL, SC, FS. All authors read and approved the final manuscript.

### Conflict of interest statement

The authors declare that the research was conducted in the absence of any commercial or financial relationships that could be construed as a potential conflict of interest.
