# Supplementary material for: Cyanobacterial Diversity in Microbial Mats from the Hypersaline Lagoon System of Araruama, Brazil: An In-depth Polyphasic Study
Source: Front Microbiol. 2017 Jun 30;8:1233. doi: 10.3389/fmicb.2017.01233 (PMC5492833; doi:10.3389/fmicb.2017.01233)
Supplement: Supplementary file 1 [file Image1.PDF]

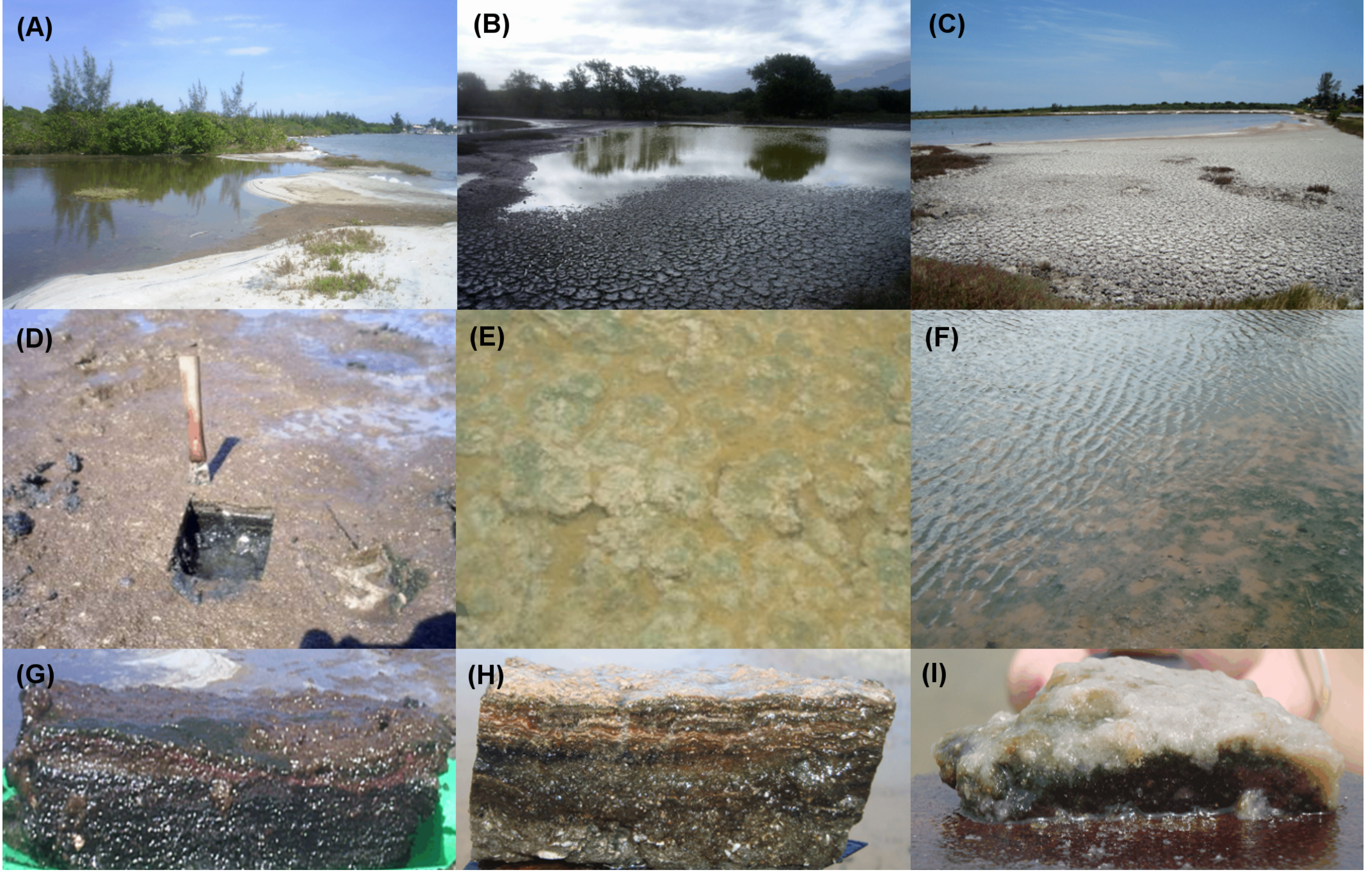

Supplementary Image S1. Sampling sites and sample mats. First columns refers to EB1 (Araruama), the second to EB2 (Pitanguinha), and the third to EB3 (Pernambuco).
(A-C) Images of the sampling sites during the dry season; the polygonal shape of the mats from EB2 and EB3 can be perceived in (B) and (C), respectively; (D-F) Cyanobacterial mats at the moment of sampling. In (D) is shown the local were the 1×1 m sample was collected, at site EB1.
(G-I) Cross section view of the samples, showing the multi-layered profile of the mats.
