# Supplementary material for: Cyanobacterial Diversity in Microbial Mats from the Hypersaline Lagoon System of Araruama, Brazil: An In-depth Polyphasic Study
Source: Front Microbiol. 2017 Jun 30;8:1233. doi: 10.3389/fmicb.2017.01233 (PMC5492833; doi:10.3389/fmicb.2017.01233)

Supplementary Image S3. Rectangular ML tree (1000 replicates) with cyanobacterial 16S rRNA gene sequences obtained in this study (marked with colored diamond squares) and their assigned phylotypes (capital letters, at right). It also includes sequences from Reference strains (white circles) and from the best BLAST hits for our sequences. Only clusters that include sequences derived from this study (i.e. obtained from 454-pyrosequencing, isolates or DGGE bands) were considered to distinguish phylotypes. Bluish diamonds are for sequences from EB1 (Araruama lagoon), greenish from EB2 (Pitanguinha), and brownish from EB3 (Pernambuco). Darker colors refer to 454-OTUs, lighter to isolates and normal colors are for DGGE-derived sequences. In bold are OTUs representing more than 4% of the total pyrosequencing reads from a sample. Additionally, it is shown the percentage value of reads for the most abundant OTU of each sample (i.e. OTUs encompassing the higher number of reads, by sample). Asterisks refer to phylotypes consisting in single sequences (i.e. loner sequences; without phylogenetically close relatives). Orange branches represent bootstrap values support >50%, and red branches >75%. Tree was rooted with the rumen Melainabacterium strain YS2 (AF544207) and *Chloroflexus aurantiacus* J-10-f (CP000909) as outgroup.

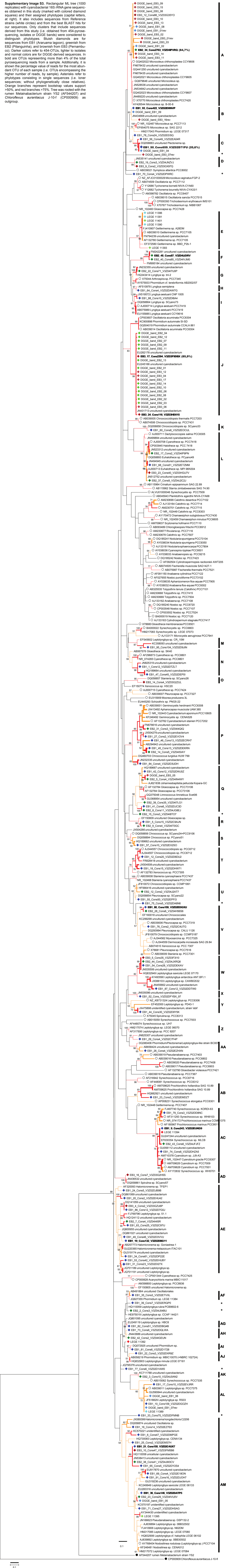

Supplement: Supplementary file 3 [file Image3.PDF]
