# Supplementary material for: Cyanobacterial Diversity in Microbial Mats from the Hypersaline Lagoon System of Araruama, Brazil: An In-depth Polyphasic Study
Source: Front Microbiol. 2017 Jun 30;8:1233. doi: 10.3389/fmicb.2017.01233 (PMC5492833; doi:10.3389/fmicb.2017.01233)
Supplement: Supplementary file 4 [file Image4.PDF]

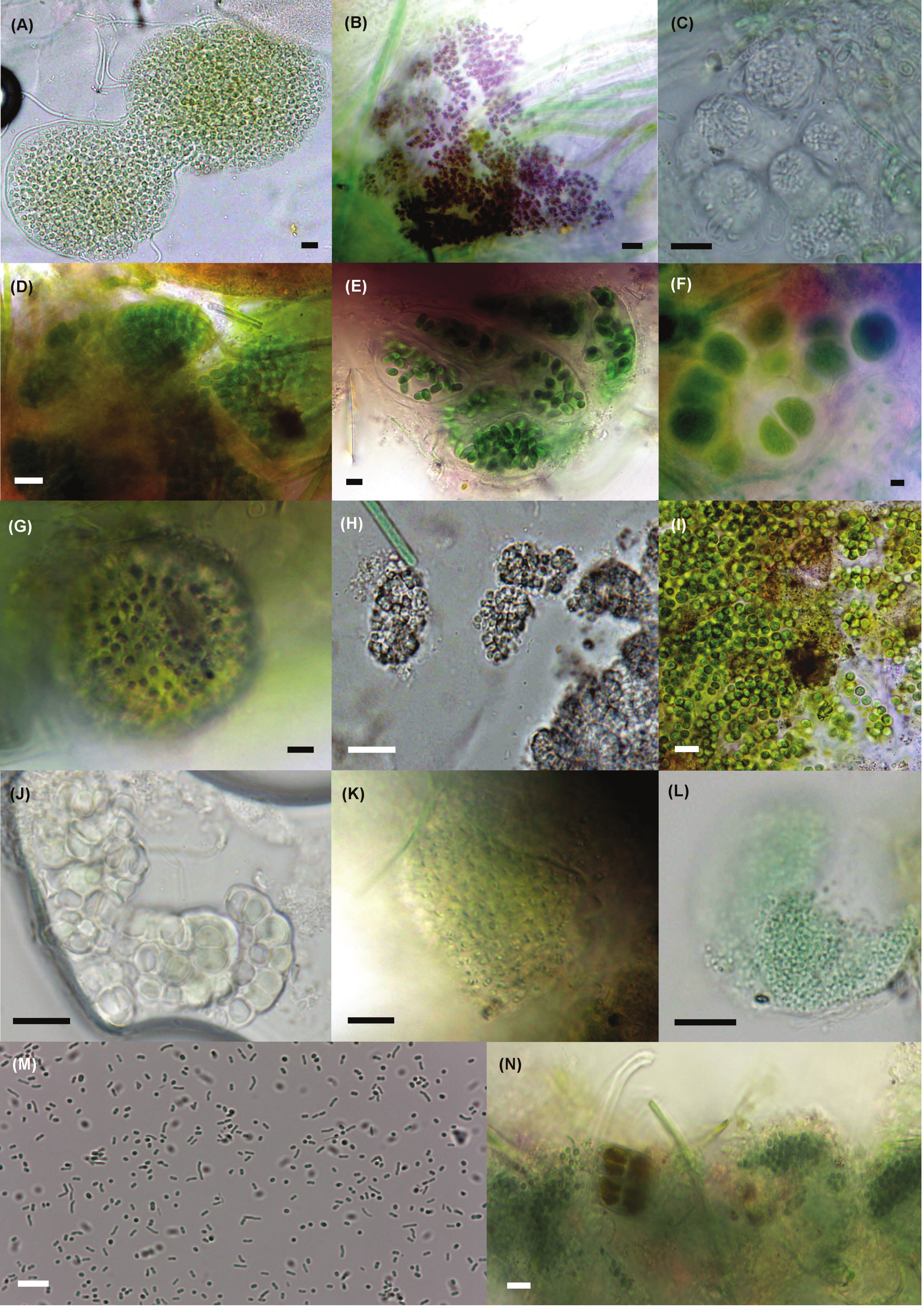

Supplementary Image S4. Light micrographs showing unicellular and colonial morphospecies from the cyanobacterial mat layers. (A) *Aphanocapsa litoralis*, (B) *Aphanocapsa* cf. *salina*, (C) *Aphanothece* cf. *conglomerata*, (D) *Aphanothece* aff. *salina*, (E) *Aphanothece* cf. *stagnina*, (F) *Chroococcus* aff. *turgidus*, (G) *Coelosphaeriopsis* cf. *halophila*, (H) *Cyanosarcina* aff. *thalassia*, (I) *Geminocystis* sp., (J) *Gloeocapsopsis crepidinum*, (K) *Gloeothece* cf. *subtilis*, (L) *Lemmermanniella* sp., (M) *Synechococcus* sp., (N) *Synechocystis salina*. Scale bar: 10 µm.
