# Supplementary material for: Cyanobacterial Diversity in Microbial Mats from the Hypersaline Lagoon System of Araruama, Brazil: An In-depth Polyphasic Study
Source: Front Microbiol. 2017 Jun 30;8:1233. doi: 10.3389/fmicb.2017.01233 (PMC5492833; doi:10.3389/fmicb.2017.01233)
Supplement: Supplementary file 5 [file Image5.PDF]

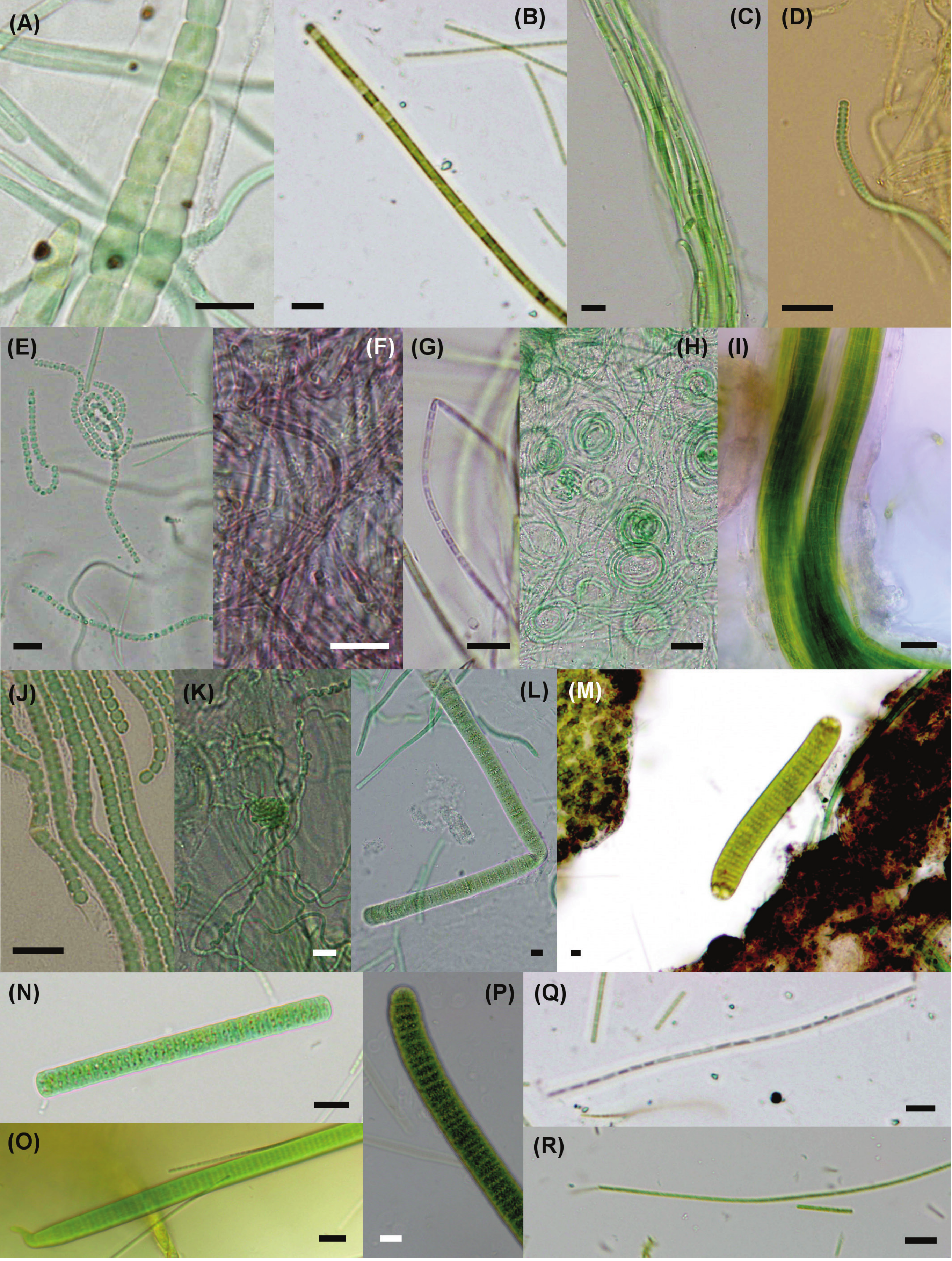

Supplementary Image S5. Light micrographs showing filamentous non-heterocystous morphospecies. (A) *Coleofasciculus chthonoplastes*, (B) *Geitlerinema* aff. *amphibium*, (C) *Geitlerinema* cf. *lemmermannii*, (D) *Komvophoron breve*, (E) *Komvophoron* cf. *minutum*, (F) *Leptolyngbya crosbyana*, (G) *Leptolyngbya* cf. *ectocarpi*, (H) *Halomicronema excentricum*, (I) *Microcoleus* aff. *steenstrupii*, (J) *Nodosilinea* sp., (K) *Nodosilinea nodulosa*, (L) *Oscillatoria limosa*, (M) *Oscillatoria margaritifera*, (N) *Oscillatoria subbrevis*, (O) *Oxynema lloydianum*, (P) *Phormidium nigroviride*, (Q) *Pseudanabaena* aff. *amphigranulata*, (R) *Pseudanabaena* cf. *limnetica*, (S) *Pseudanabaena* cf. *minima*, (T) *Spirulina labyrinthiformis*. Scale bar: 10 µm.
