# Supplementary material for: Cyanobacterial Diversity in Microbial Mats from the Hypersaline Lagoon System of Araruama, Brazil: An In-depth Polyphasic Study
Source: Front Microbiol. 2017 Jun 30;8:1233. doi: 10.3389/fmicb.2017.01233 (PMC5492833; doi:10.3389/fmicb.2017.01233)
Supplement: Supplementary file 6 [file Image6.PDF]

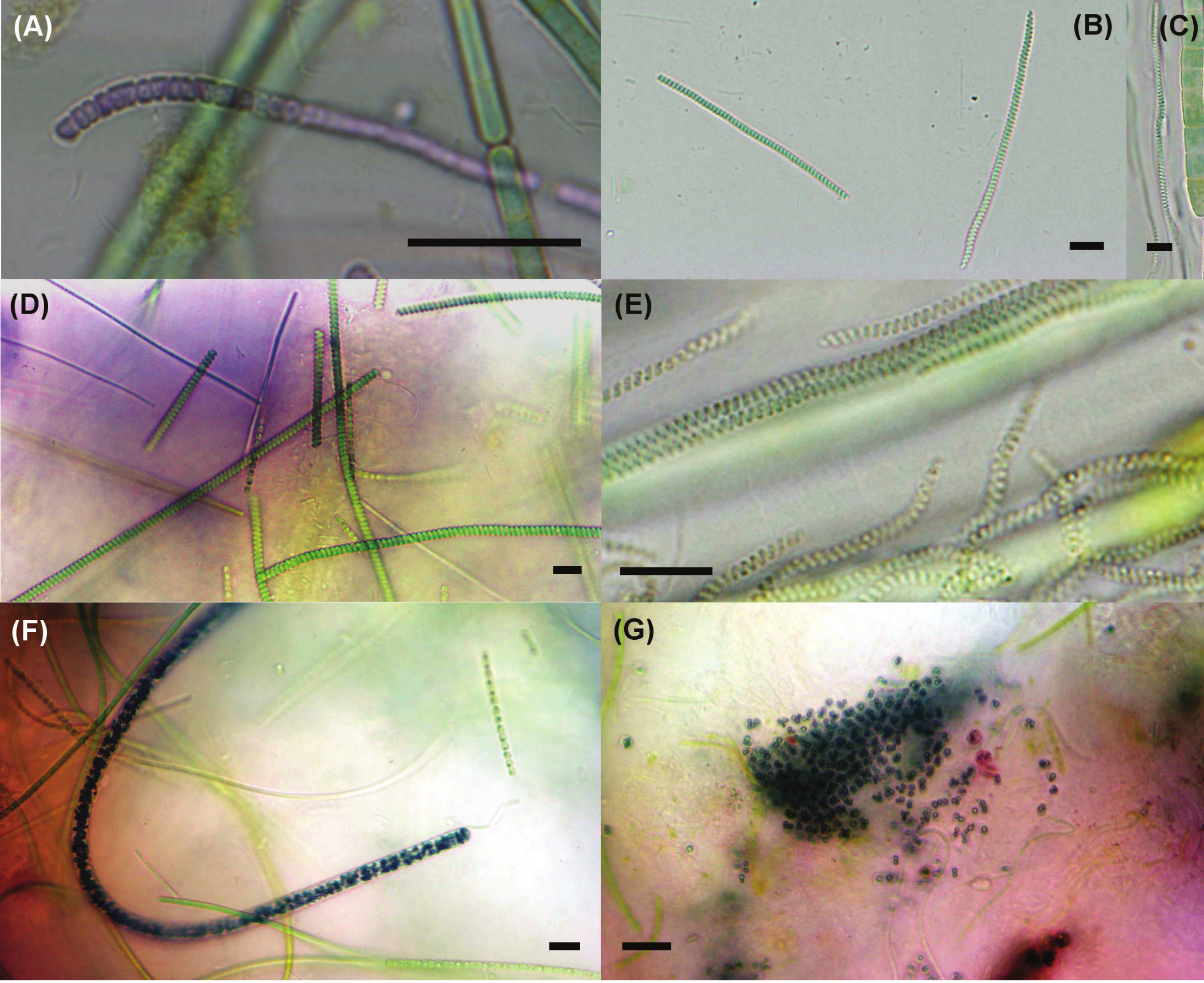

Supplementary Image S6. Light micrographs showing filamentous non-heterocystous Cyanobacteria (A-E) and Proteobacteria (F-G). (A) *Pseudanabaena* cf. *minima*, (B) *Spirulina labyrinthiformis*, (C) *S. labyrinthiformis* embedded within the sheath of *Coleofasciculus chthonoplastes*, (D) *Spirulina subsalsa*, (E) *Spirulina tenerrima*, (F) the motile, colorless, filamentous sulfur bacteria *Beggiatoa* sp., (G) a species of purple sulfur bacteria from the family Chromatiaceae. Scale bar: 10 μm.
