# Supplementary material for: Cyanobacterial Diversity in Microbial Mats from the Hypersaline Lagoon System of Araruama, Brazil: An In-depth Polyphasic Study
Source: Front Microbiol. 2017 Jun 30;8:1233. doi: 10.3389/fmicb.2017.01233 (PMC5492833; doi:10.3389/fmicb.2017.01233)
Supplement: Supplementary file 7 [file Image7.PDF]

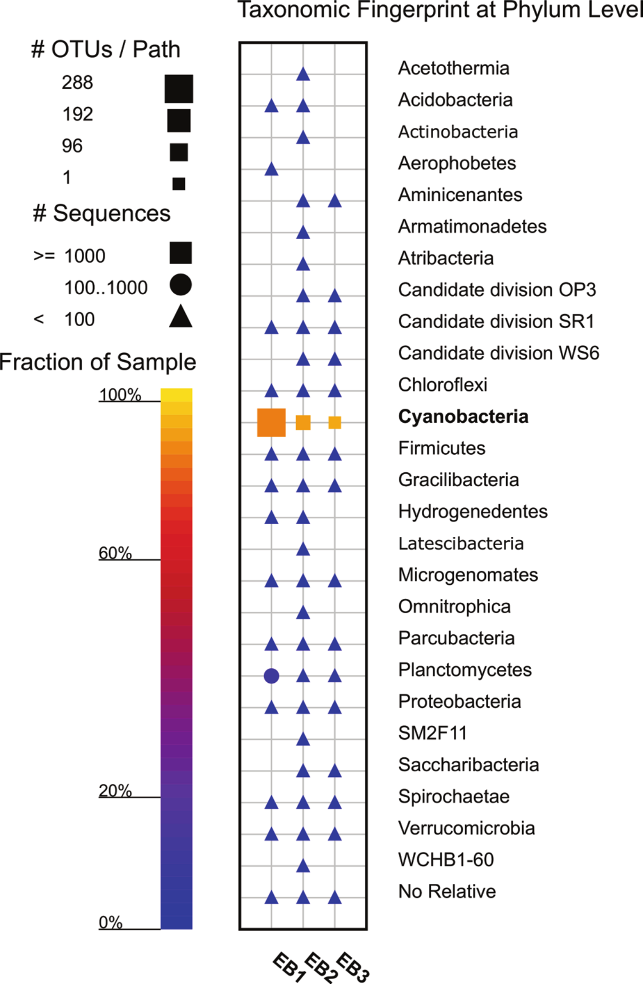

Supplementary Image S7. Number and relative abundance of OTUs (clustered at 98% similarity) generated by the SILVAngs pipeline, at the phylum level.
