# Supplementary material for: Cyanobacterial Diversity in Microbial Mats from the Hypersaline Lagoon System of Araruama, Brazil: An In-depth Polyphasic Study
Source: Front Microbiol. 2017 Jun 30;8:1233. doi: 10.3389/fmicb.2017.01233 (PMC5492833; doi:10.3389/fmicb.2017.01233)
Supplement: Supplementary file 9 [file Image9.PDF]

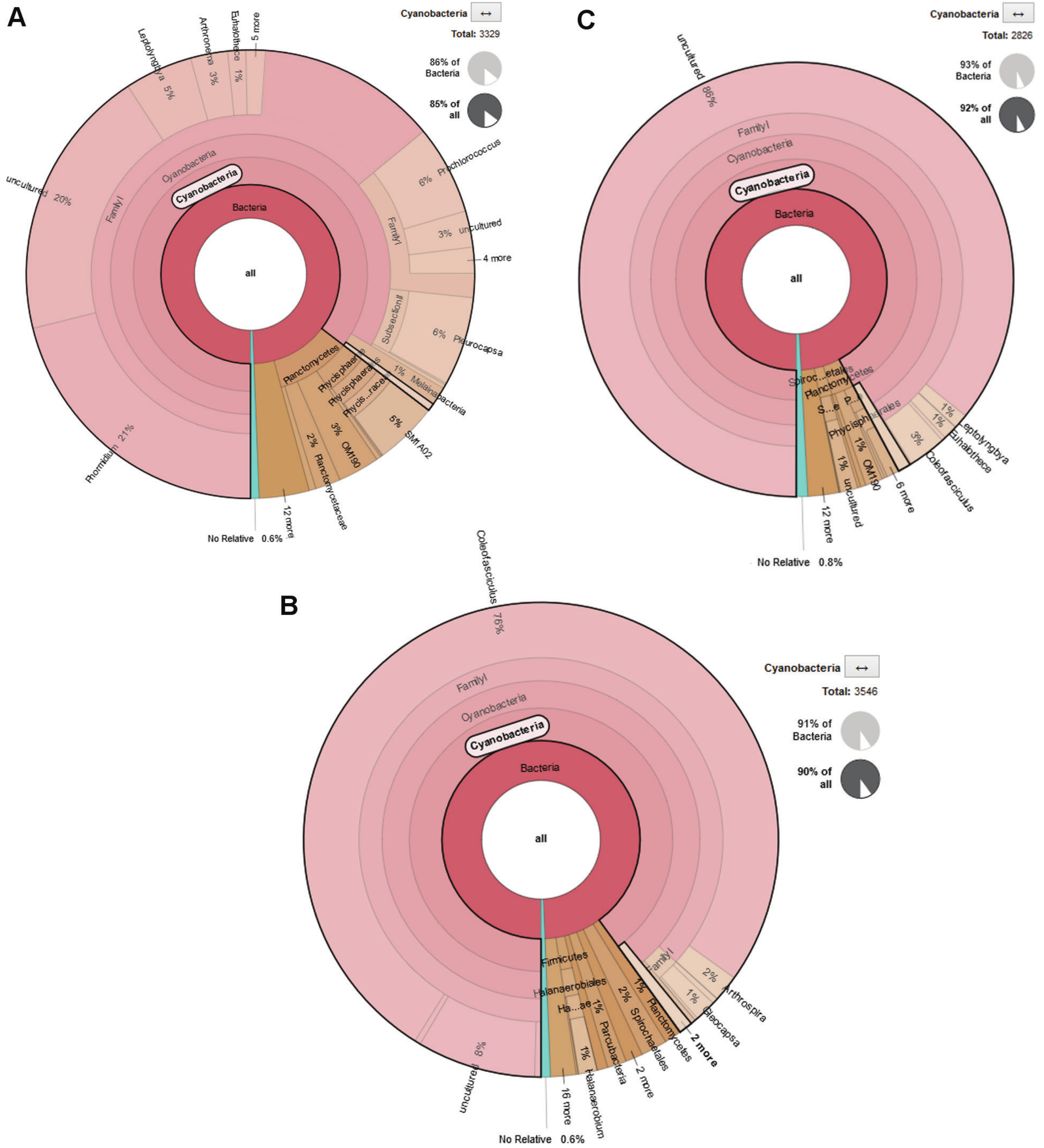

Supplementary Image S9. Krona charts screenshots illustrating the metagenomics results for the mat samples collected at EB1 (A), EB2 (B) and EB3 (C). The interactive version of these pie charts can be accessed at http://www.webcitation.org/6kiUALfVA.
