# Supplementary material for: Cyanobacterial Diversity in Microbial Mats from the Hypersaline Lagoon System of Araruama, Brazil: An In-depth Polyphasic Study
Source: Front Microbiol. 2017 Jun 30;8:1233. doi: 10.3389/fmicb.2017.01233 (PMC5492833; doi:10.3389/fmicb.2017.01233)

**Supplementary Image S10.** Circular phylogenetic ML tree based on cyanobacterial 16S rDNA sequences; labelled version of Figure 6. Sequences obtained in this study are marked with colored diamond squares (see below), while white circles denote sequences from Reference strains. Tree branches in orange represent values of bootstrap support >50%, and in red >75%.

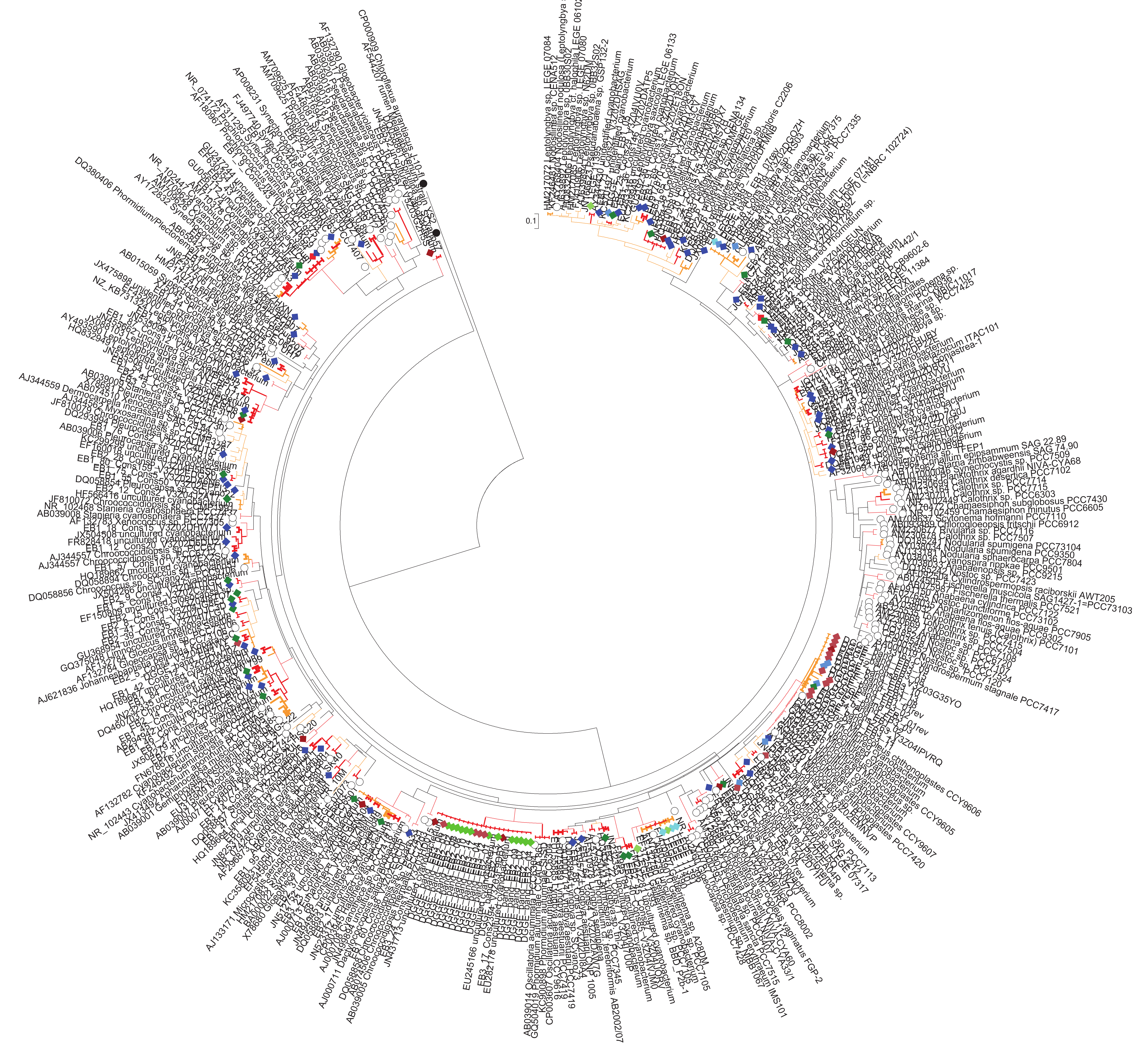

Supplement: Supplementary file 10 [file Image10.PDF]
