# Supplementary material for: Cyanobacterial Diversity in Microbial Mats from the Hypersaline Lagoon System of Araruama, Brazil: An In-depth Polyphasic Study
Source: Front Microbiol. 2017 Jun 30;8:1233. doi: 10.3389/fmicb.2017.01233 (PMC5492833; doi:10.3389/fmicb.2017.01233)
Supplement: Supplementary file 11 [file Image11.PDF]

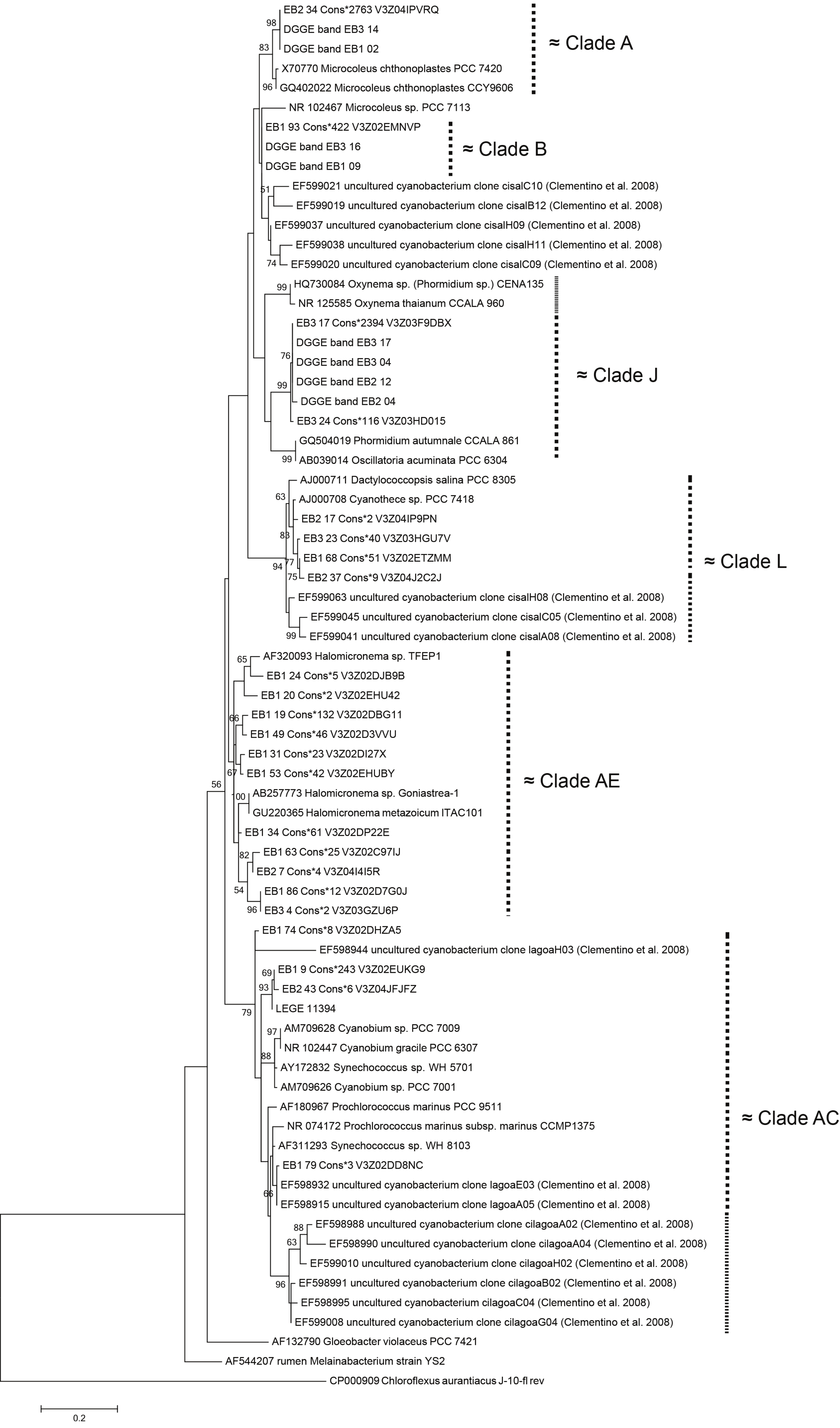

Supplementary Image S11. Complementary ML tree based on a selection of cyanobacterial 16S rDNA sequences from this study (with indication of the respective clade; see Supplementary Image S2), from the study of Clementino et al. (2008) and from *Oxynema* spp. (Chatchawan et al., 2012). The analysis involved 71 nucleotide sequences. Values of bootstrap support >50% are shown near the nodes. Due to sequence shortness and/or low overlapping, fewer than 6% alignment gaps, missing data, and ambiguous bases were allowed at any position. There were a total of 203 positions in the final dataset.
