# Supplementary material for: Cyanobacterial Diversity in Microbial Mats from the Hypersaline Lagoon System of Araruama, Brazil: An In-depth Polyphasic Study
Source: Front Microbiol. 2017 Jun 30;8:1233. doi: 10.3389/fmicb.2017.01233 (PMC5492833; doi:10.3389/fmicb.2017.01233)
Supplement: Supplementary file 12 [file Table1.PDF]

Supplementary Material

Cyanobacterial diversity in microbial mats from the hypersaline coastal lagoon system of Araruama, Brazil: an in-depth polyphasic approach

Vitor Ramos[1,2], Raquel Castelo-Branco[1], Pedro Leão[1], Joana Martins[1,2], Sinda Carvalhal-Gomes[3], Frederico Sobrinho da Silva[3], João Graciano Mendonça Filho[3], Vitor Vasconcelos[1,2,*]

1 Faculty of Sciences, University of Porto, Porto, Portugal

2 Interdisciplinary Centre of Marine and Environmental Research (CIIMAR/CIMAR), University of Porto, Matosinhos, Portugal

3 Palynofacies & Organic Facies Laboratory, Department of Geology, Federal University of Rio de Janeiro, Brazil

*** Correspondence:** Vitor Vasconcelos: vmvascon@fc.up.pt

Supplementary Table S1. Checklist of cyanobacteria species reported for different water bodies from the Araruama lagoon system, as depicted from this study (highlighted **in bold**) or retrieved from the literature survey.

Taxa[# +]	Sample description			Lagoon[£]	References
	Type	Microbial structure[*]	Shape[§]		
**Chroococcales**					
Aphanothece castagnei (Brébisson) Rabenhorst	mat, sediment, rock	l, lay, s	b, colu, d, f, po, pu, sm, t	ARA (man-made saltern), BES, BPF, PER, PIT, SAJ	1-4, 6, 8, 17
Aphanothece clathrata West & G.S.West **(17)**	mat, rock	l, lay, non	b, f, po, pu, sm, t	ARA (man-made saltern), BES, PER	2, 4, 17
***Aphanothece conglomerata* Rich**	mat, sediment, rock, water	l, lay, non	coll, f, sm	**ARA**, ARA (man-made saltern), BES, BPF, PER, PIT	1, 4, 6, 7, 10, 17, **this study**
Aphanothece halophytica Frémy **(6)(7)**	mat, sediment, rock, water	l, lay, s, t	b, coll, colu, d, f, po, pu, sm, t	ARA (man-made saltern), BES, BPF, PER, PIT, SAJ	1-8, 10-12, 15, 17
Aphanothece marina (Ercegovic) Komárek & Anagnostidis	mat, sediment, rock	l, lay	b, coll, f, po, pu, sm, t	ARA (man-made saltern), BES, PER, PIT	1-4, 10, 11, 15, 17
Aphanothece microscopica Nägeli	mat, water	lay	f, sm	ARA (man-made saltern), BES, PIT	7, 17
Aphanothece pallida (Kützing) Rabenhorst	mat, rock, water	l, non	b, coll, f, po, pu, t	PIT	1-4, 7, 10
***Aphanothece salina* Elenkin & Danilov**	mat, sediment, rock, water	l, lay, non	coll, f, po, pu, sm	**ARA**, ARA (man-made saltern), BPF, PER, PIT, SAJ	1, 3, 4, 6-8, 11, 17, **this study**
Aphanothece saxicola Nägeli	mat, sediment, rock	l, lay, s, t	b, colu, d, f, po, pu, sm, st, t	ARA (man-made saltern), BES, BPF, PER, PIT	1-6, 10-12, 15, 17
***Aphanothece stagnina* (Sprengel) A.Braum (6) (17)**	mat, sediment, rock, water	l, lay, s	b, coll, f, po, pu, sm, t	**ARA**, ARA (man-made saltern), BES, BPF, **PER**, **PIT**, SAJ	1-8, 10, 14, 17, **this study**
Chlorogloea tuberculosa (Hansgirg) Wille	mat	lay	f, sm	BES	17
Chroococcus sp. **(17)**	mat	lay	f, sm	BES	17
Chroococcus dispersus (Keissler) Lemmermann	mat, sediment, rock	l, lay	coll, f, po, sm	PER, PIT	1, 3, 4, 11, 15
Chroococcus giganteus West	mat, rock	l, lay	f, po, sm	PER, PIT	1, 3, 4, 11
Chroococcus membraninus (Meneghini) Nägeli	mat, rock, water	l, lay, s, t	b, coll, f, po, sm	AZU, BES, BPF, PER, PIT	1, 3-5, 7, 9-12, 14, 15, 17
Chroococcus microscopicus J.Komárková-Legnerová & G.Cronberg **(8)**	mat, sediment, rock	l, lay, t	b, coll, f, po, pu, sm	ARA (man-made saltern), BES, BPF, PER, PIT, SAJ	1, 3-6, 8, 10, 12, 14, 15, 17

Chroococcus minimus (Keissler) Lemmermann **[8] [17]**	mat, sediment, rock, water	l, lay, t	b, coll, f, po, pu, sm	ARA (man-made saltern), BES, BPF, PER, PIT, SAJ	1, 3, 4, 6-8, 10-12, 14, 15, 17
Chroococcus minor (Kützing) Nägeli	mat, sediment, rock	l, lay, s, t	b, coll, colu, d, f, po, pu, sm, st, t	ARA (man-made saltern), AZU, BES, BPF, PER, PIT	1-4, 6, 9-12, 14, 15, 17
Chroococcus minutus (Kützing) Nägeli **[4] [17]**	mat, sediment, rock, water	l, lay, t	b, coll, f, po, pu, sm	ARA (man-made saltern), BES, BPF, PER, PIT, SAJ	1, 3-8, 10-12, 14, 15, 17
Chroococcus obliteratus Richter	mat, rock	l, non	coll, po, sm	PER, PIT	1, 3, 4, 10
Chroococcus prescottii Drouet & Daily	mat	non	b, coll	BPF, PIT	1, 5, 10
Chroococcus quaternarius Zalessky	mat, sediment, rock	l, lay	coll, f, po, sm	AZU, PER, PIT	1, 3, 4, 9, 11
Chroococcus submarinus (Hansgirg) Kováčik	sediment	-	-	AZU	9
Chroococcus tenax (Kirchner) Hieronymus	mat	lay	coll, f, sm	BES, PIT	10, 11, 14
***Chroococcus turgidus* (Kützing) Nägeli [4][8]**	mat, sediment, rock, water	l, lay, s	b, coll, colu, d, f, po, pu, sm, st	**ARA**, ARA (man-made saltern), AZU, BES, BPF, PER, PIT, SAJ	2-6, 8-12, 14, 15, 17, **this study**
Chroococcus turicensis (Nägeli) Hansgirg	water	-	-	PIT	7
***Cyanosarcina thalassia* Anagnostidis & Pantazidou**	mat, rock	l, lay, t	coll, f, po, pu, sm	BES, PER, **PIT**	1, 3, 4, 11, 12, 14, 17, **this study**
Entophysalis conferta (Kützing) Drouet & Daily **[1]**	mat	lay	b, f, po, pu, sm, t	BES, PIT	1, 11, 14, 17
Entophysalis granulosa Kützing **[1][4][11]**	mat, rock	l, lay	coll, f, po, pu, sm	BES, PER, PIT	1, 3, 4, 10, 11, 14, 17
***Geminocystis* sp.**	mat	lay	po, sm	**ARA**, **PER**, **PIT**	**this study**
Gloeocapsa sp.	mat, rock	stromatolitic -like, lay	-	VER	16
Gloeocapsa punctata Nägeli	mat	lay	b, f, po, pu, t	PIT	1, 11
***Gloeocapsopsis* cf. *crepidinum* (Thuret) Geitler ex Komárek**	mat	lay	b, f, po, pu, sm, t	**ARA**, BES, **PIT**	1, 17, **this study**
Gloeocapsopsis magma (Brébisson) Komárek & Anagnostidis	mat	lay	b, f, po, pu, sm, t	ARA (man-made saltern), PIT	1, 11, 17
Gloeothece confluens Nägeli	mat, rock	l, non	b, coll, f, po, pu, sm, t	PER, PIT, SAJ	1, 3, 4, 8, 10
Gloeothece linearis Nägeli	mat, rock	l, lay	b, coll, f, po, pu, sm, t	BES, PER, PIT	1, 3, 4, 11, 17
***Gloeothece subtilis* Skuja**	mat, sediment	lay	b, f, po, pu, sm, t	**ARA**, PIT	1, 11, 15, **this study**
Gloeothece vibrio N.Carter **[8]**	mat	lay, non	b, f, po, pu, sm, t	ARA (man-made saltern), PIT, SAJ	1, 8, 17
Gomphosphaeria aponina Kützing	mat, rock	l, non	coll, po, sm	PER	3, 4
Halothece sp. (including "*Euhalothece*" *sensu* Garcia-Pichel *et al.*(1998); 11 OTUs)**[&]**	water	-	-	ARA	13

Johannesbaptistia pellucida (Dickie) W.R.Taylor & Drouet	mat, sediment, rock	l, lay	b, coll, f, po, pu, sm, t	AZU, BES, PER, PIT	1-4, 9, 14, 15, 17
Lithocapsa fasciculata Ercegovic	mat	non	b, f, po, pu, t	PIT	1
Pseudocapsa dubia Ercegovic	mat	lay	f, sm	BES, PIT	1, 11, 14
Pseudocapsa maritima Komárek	mat	lay	f, sm	BES	17
Pseudocapsa sphaerica (Proskina-Lavrenko) Kovácik	mat	lay	b, f, po, pu, sm, t	ARA (man-made saltern), BES, PIT, SAJ	1, 8, 11, 14, 17
Xenococcus schousboei Thuret	mat, sediment	lay	b, f, po, pu, sm, t	PIT	1, 11, 15
Xenotholos kerneri (Hansgirg) M.Gold-Morgan *et al.*	mat	lay	b, f, po, pu, sm, t	ARA (man-made saltern), PIT, SAJ	1, 8, 11, 17

Chroococcidiopsidales

Chroococcidiopsis fissurarum (Ercegovic) Komárek & Anagnostidis	water	-	-	PIT	7

Oscillatoriales

Coleofasciculus (=*Microcoleus*) ***chthonoplastes* (Gomont) M.Siegesmund *et al*.** [(1)(2)(3)(4)(10)]	mat, sediment, rock, water	l, lay, s	b, coll, colu, d, f, po, pu, sm, st, t	ARA (man-made saltern), AZU, BES, **PER**, **PIT**, SAJ	1-4, 7-10, 14, 15, 17, **this study**
***Geitlerinema* aff. *amphibium* (Agardh ex Gomont) Anagnostidis**	mat	lay	po	**PIT**	**this study**
***Geitlerinema* cf. *lemmermannii* (Woloszynska) Anagnostidis**	mat	lay	po, sm	**ARA**, **PER**, **PIT**	**this study**
Lyngbya aestuarii Liebman ex Gomont [(1)(4)(10)(14)]	mat, sediment, rock	l, lay, s	b, coll, colu, d, f, po, pu, sm, st, t	BES, BPF, PER, PIT	1-4, 6, 10, 14
Lyngbya confervoides Agardh ex Gomont	mat	non	b, f, po, pu, t	PER	2
Lyngbya (=*Porphyrosiphon*) *martensianus* Meneghini ex Gomont	Mat	lay	sm	BES	14
***Microcoleus* aff. *steenstrupii* Petersen**	Mat	lay	po, sm	**ARA**, **PER**	**this study**
Microcoleus vaginatus Gomont ex Gomont	mat, rock, water	l, non, s	b, coll, colu, d, f, po, pu, sm, st, t	PER, PIT, SAJ	1-4, 7, 8, 11
Microcoleus sp.	mat, rock	stromatolitic -like, lay	-	VER	16
***Oscillatoria limosa* Agardh ex Gomont**	Mat	lay, non	b, f, po, pu, t	**PER**, **PIT**	2, **this study**
***Oscillatoria margaritifera* Kützing ex Gomont**	Mat	lay	po	**PIT**	**this study**
Oscillatoria (=*Spirulina*) *meneghiniana* Zanardini ex Gomont	mat, water	non	b	BPF, PIT	5, 7
***Oscillatoria subbrevis* Schmidle**	mat, sediment, rock	l, non	b, coll, f, po, pu, sm, t	AZU, PER, **PIT**	2-4, 9, **this study**

Oscillatoria terebriformis f. *amphigranulata* Elenkin & Kossinskaja	mat	non	b, f, po, pu, t	PER	2
Oscillatoria sp.	mat	lay	f, sm	ARA (man-made saltern)	17
Oxynema (=*Phormidium*) *acuminatum* (Gomont) Chatchawan *et al.*	mat, rock	l, lay	b, coll, f, po, pu, sm, t	BES, PER, PIT	1, 3, 4, 11, 14, 17
***Oxynema lloydianum* (Gomont) Chatchawan, Komárek, Strunecky, Smarda & Peerapornpisal**	mat	lay	po, sm	**PER**, **PIT**	**this study**
Phormidium acutum (Brühl & Biswas) Anagnostidis & Komárek	mat, rock	l, non	b, coll, f, po, pu, sm, t	PER, PIT	1, 3, 4
Phormidium articulatum (=*Oscillatoria articulata*) (Gardner) Anagnostidis & Komárek	mat	Non	b, f, po, pu, t	PER	2
Phormidium breve (Kützing ex Gomont) Anagnostidis & Komárek	mat, sediment, rock	l, lay, t	b, coll, f, po, pu, sm, t	AZU, BPF; PER, PIT	1, 3, 4, 6, 9, 11, 12
Phormidium (=*Oscillatoria*) *foreaui* (Frémy) Umezaki & Watanabe	water	-	-	PIT	7
Phormidium formosum (Bory ex Gomont) Anagnostidis & Komárek	mat, sediment	Non	b, f, po, pu, t	AZU, PER, PIT	1, 3, 4, 9
Phormidium hamelii (Frémy) Anagnostidis & Komárek	mat, rock	l, lay	b, coll, f, po, pu, sm, t	PER, PIT	1, 3, 4, 11
Phormidium hormoides Setchell & Gardner	mat, rock	l, non	b, coll, f, po, pu, sm, t	PER	3, 4
Phormidium minnesotense (Tilden) Drouet	mat	non	po	PER	3, 4
***Phormidium nigroviride* (Thwaites ex Gomont) Anagnostidis & Komárek**	mat	lay	po	**PIT**	**this study**
Phormidium okenii (Agardh) Anagnostidis & Komárek [(4)]	mat, sediment, rock	l, lay, t	b, f, po, pu, sm, t	ARA (man-made saltern), AZU, BES, PER, PIT	1, 3, 4, 9, 11, 12, 14, 17
Phormidium terebriforme (Agardh ex Gomont) Anagnostidis & Komárek	mat, rock	l, non	b, f, po, pu, sm, t	PER	1, 3, 4
Phormidium willei (Gardner) Anagnostidis & Komárek [(1)]	mat, rock	l, non, t	b, coll, f, po, pu, sm, t	PIT	1, 3, 4, 12
Planktothrix rubescens (De Candolle ex Gomont) Anagnostidis & Komárek	rock	L	sm	PER	4
Symplocastrum (=*Schizothrix*) *friesii* (Gomont ex Gomont) Kirchner [(1)(2)(4)(14)]	mat, sediment, rock	l, lay, s, t	b, coll, f, po, pu, sm, t	ARA (man-made saltern), BES, PER, PIT, SAJ	1-4, 8, 10-12, 14, 15, 17
Trichodesmium lacustre (=*Oscillatoria lacustris*) (Klebahn) Geitler	mat	lay	b, f, po, pu, t	PIT	1, 11
unidentified Oscillatoriales (*Coleofasciculus*-related, 87-93%	water	-	-	ARA	13

similarity; 8 OTUs)[&]					
**Nostocales**					
Brachytrichia quoyi Bornet & Flahault	mat, rock	non, s	b, colu, d, f, po, pu, st, t	PER	2
Calothrix crustacea Schousboe ex Thuret	mat, rock	non, s	b, colu, d, f, po, pu, st, t	PER	2
Calothrix scopulorum (Weber & Mohr) Agardh	rock	l	sm	PER	4
Kyrtuthrix maculans (Gomont) Umezaki	mat, sediment, rock	l, lay	b, coll, f, po, pu, sm, t	AZU, PER, PIT	1, 3, 4, 9, 11, 15
**Pleurocapsales**					
Pleurocapsa fuliginosa Hauck	mat, rock	lay, t	f	PER, PIT	1, 11, 12
**Spirulinales**					
***Spirulina labyrinthiformis* Gomont**	mat	lay	po	**PER**	**this study**
Spirulina laxissima G.S.West	sediment	-	-	BPF	6
Spirulina meneghiniana Zanardini ex Gomont	mat	lay	f, sm	BES	17
***Spirulina subsalsa* Oerstedt ex Gomont** **(6)(8)**	mat, sediment	l, lay, non	b, coll, f, po, pu, sm, t	ARA (man-made saltern), BES, BPF; **PER**, PIT, SAJ	1, 2, 4, 6, 8, 10, 17, **this study**
Spirulina subtilissima Kützing ex Gomont	mat, sediment, rock	l, lay, s	b, coll, f, po, pu, sm, t	BES, PER, PIT, SAJ	1-4, 8, 10, 11, 14, 15, 17
***Spirulina tenerrima* Kützing ex Gomont**	mat	lay	po	**PER**	**this study**
Spirulina sp.	mat, rock	stromatolitic -like, lay	-	VER	16
**Synechococcales**					
***Aphanocapsa litoralis* (Hansgirg) Komárek & Anagnostidis**	mat	l, lay	pu, sm	**ARA**, BES, PER, PIT, SAJ	1, 4, 8, 17, **this study**
Aphanocapsa rivularis (Carmichael) Rabenhorst	mat	l, lay	f, sm	BES	17
***Aphanocapsa salina* Woronichin**	mat	lay	po, pu, sm	**ARA**, **PIT**, SAJ	8, **this study**
Aphanocapsa salinarum Hansgirg	-	-	-	PIT	1
Bacularia caerulescens Borzì	mat, rock, water	lay	f	PIT	1, 7, 11, 15
***Coelosphaeriopsis* cf. *halophila* Lemmermann**	mat	lay	sm	**ARA**	**this study**
***Halomicronema excentricum* Abed, Garcia-Pichel & Hernández-Mariné** [&]	mat	lay	po, sm	**ARA**, **PER**, **PIT**	**this study**

Jaaginema subtilissimum (=*Oscillatoria subtilissima*) (Kützing ex De Toni) Anagnostidis & Komárek	mat, water	lay	f, sm	BES, PIT	7, 17
***Komvophoron breve* (Carter) Anagnostidis (subgenus *Alyssophoron* Anagnostidis et Komárek 1988)**	mat	lay	sm	**ARA**	**this study**
***Komvophoron* cf. *minutum* (Skuja) Anagnostidis & Komárek**	mat	lay	po	**PER**	**this study**
***Lemmermanniella* sp. Geitler in Engler & Prantl**	mat	lay	sm	**ARA**	**this study**
***Leptolyngbya crosbyana* (Tilden) Anagnostidis & Komárek**	mat	lay	po, sm	**ARA**, **PIT**	**this study**
***Leptolyngbya* cf. *ectocarpi* (Gomont) Anagnostidis & Komárek**[% &]	mat	lay	sm	**ARA**	**this study**
Leptolyngbya (=*Lyngbya*) *fragilis* (Gomont) Anagnostidis & Komárek **(1)**	mat, sediment, rock	lay, t	coll, po, sm	BES, BPF, PER, PIT	1, 3, 4, 6, 10, 12, 14
Leptolyngbya hypolimnetica (=*Phormidium hypolimneticum*) (Campbell) Anagnostidis **(17)**	mat, rock	l, lay, non	coll, f, po, pu, sm	ARA (man-made saltern), BES, PER	3, 4, 17
Leptolyngbya komarovii (Anissimova) Anagnostidis & Komárek	mat, rock	l, lay, s	b, coll, colu, d, f, po, pu, sm, st, t	BES, PER, PIT	1-4, 14
Leptolyngbya (=*Lyngbya*) *submonilifera* (Frémy) Senna & Compère	Mat	lay	f, sm	BES	17
Leptolyngbya tenuis (Gomont) Anagnostidis & Komárek **(17)**	mat, sediment, rock	l, lay	coll, f, po, sm	ARA (man-made saltern), AZU, BES, PER, PIT	1, 3, 4, 9-11, 14, 15, 17
Leptolyngbya sp. **(17)**	mat	lay	f, sm	BES	17
Limnococcus (=*Chroococcus*) *limneticus* (Lemmermann) Komárková *et al.*	mat	non	b, f, po, pu, t	PER	2
Merismopedia warmingiana Lagerheim	mat	non	b	BPF	5
***Nodosilinea nodulosa* (Li & Brand) Perkerson & Casamatta**	Mat	lay	sm	**ARA**	**this study**
***Nodosilinea* sp.** [% &]	Mat	lay	po	**PIT**	**this study**
Planktolyngbya limnetica (=*subtilis*) (Lemmermann) J.Komárková-Legnerová & G.Cronberg	mat, sediment, rock	l, lay	b, f, po, pu, sm, t	BES, BPF; PER, PIT	1, 4, 6, 14, 17
***Pseudanabaena* aff. *amphigranulata* (Goor) Anagnostidis**	mat	lay	po	**PIT**	**this study**
Pseudanabaena (=*Oscillatoria*) ***limnetica* (Lemmermann) Komárek**	mat, rock	l, non	b, coll, f, po, pu, sm, t	**ARA**, PER, PIT	1, 3, 4, **this study**

***Pseudanabaena minima* (G.S.An) Anagnostidis**	mat	lay	sm	**ARA**	**this study**
Schizothrix arenaria Gomont	mat, rock	l, lay	b, coll, f, po, pu, sm, t	BES, PER, PIT	1, 3, 4, 14
Synechococcus elongatus (Nägeli) Nägeli	mat, sediment, rock	l, lay, t	b, coll, f, po, pu, sm, t	AZU, BES, BPF; PER, PIT	1, 3, 4, 6, 9, 11, 12, 14, 15, 17
Synechococcus salinarum Komárek	mat, rock	l, non	b, f, po, pu, sm, t	PER, PIT, SAJ	1, 4, 8
Synechococcus subsalsus Skuja	water	-	-	PIT	7
Synechococcus sp.	mat	lay	f, sm	BES	17
***Synechococcus* sp.** [%, &]	mat	lay	po	**PER**	**this study**
Synechococcus sp. (6 OTUs)[&]	water	-	-	ARA	13
***Synechocystis salina* Wislouch**	mat, sediment	lay	sm	**ARA**, BPF	6, **this study**
Trichocoleus (=*Microcoleus*) *tenerrimus* (Gomont) Anagnostidis [(4)]	mat, rock, water	l, non, s	b, coll, colu, d, f, po, pu, sm, st, t	PER, PIT	2-4, 7

\# Numbers in brackets are bibliographic references of studies indicating that the species was dominating/was in high abundance in the sample (qualitative estimates).

\+ Species names are currently accepted taxonomic entities according to Guiry and Guiry [18] and higher taxonomic ranks are according to the most recent system of classification for cyanobacteria, proposed by Komárek *et al.* [19] (see also Material and Methods section; main text). Older synonyms used in the consulted references are also shown (in brackets)

& Phylotypes or operational taxonomic units (OTUs) identified/characterized by 16S rRNA gene sequencing.

% Organism (also) detected by culture-dependent methodology. See Table 4 for a list of isolates obtained in this study and Table 3 and Figure 5 for their characterization.

* l, laminites; lay, layered organic (i.e. non-lithifying) mat; non, organic (i.e. non-lithifying) mat; s, stromatolites; t, thrombolites

§ b, blistered; coll, colloform; colu, columnar; d, domal; f, flat; po, polygonal; pu, pustular; sm, smooth; st, stratiform; t, tufted

£ Abbreviations for the lagoons: **ARA**, Araruama; AZU, Azul; BES: Brejo do Espinho; BPF, Brejo do Pau Fincado; **PER**, Pernambuco; **PIT**, Pitanguinha; SAJ, Salina Julieta; VER, Vermelha. Studied water bodies (EB1 is located in **ARA**; EB2 → **PIT**; EB3 → **PER**) are highlighted in bold if the organism was observed in samples from this work and are underlined if the observation corresponds to a first report (see Table 3).

References:

1. Damazio, C.M. *et al.*, 2005. *Geociências* 10 (6): 11-16. [in Portuguese]

2. Silva, L.H.S. *et al.*, 2004. *Revista Brasileira de Paleontologia* 7(2): 189-192. [in Portuguese]

3. Iespa, A.A.C. *et al.*, 2009. *Journal of Geoscience* 5(1): 35-41.

4. Iespa, A.A.C. *et al.*, 2005. *Geociências* 10 (6): 5-10. [in Portuguese]

5. Silva, L.H.S. *et al.*, 2007. *Anuário do Instituto de Geociências* 30(1): 188-193. [in Portuguese]

6. Silva, L.H.S. *et al.*, 2011. *Anuário do Instituto de Geociências* 34(2) 14-23. [in Portuguese]

7. Silva, L.H.S. *et al.*, 2007. *Revista Biociências* 13(1-2): 63-70. [in Portuguese]

8. Silva, L.H.S. *et al.*, 2007. *Anuário do Instituto de Geociências* 30(1): 175-180. [in Portuguese]

9. Silva, L.H.S. *et al.*, 2008. *Anuário do Instituto de Geociências* 31(1): 24-29. [in Portuguese]

10. Damazio *et al.*, 2006. *Revista Brasileira de Paleontologia* 9(1):165-170. [in Portuguese]

11. Silva, L.H.S. *et al.*, 2007 *Anuário do Instituto de Geociências* 30(1): 67-72. [in Portuguese]

12. Silva, L.H.S. *et al.*, 2006 *Revista de Biologia e Ciências da Terra* 6(2): 243-250. [in Portuguese]

13. Clementino, M.M. *et al.*, 2008 *Extremophiles* 12: 595-604.

14. Silva, L.H.S. *et al.*, 2007 *Anuário do Instituto de Geociências* 30(1): 181-187. [in Portuguese]

15. Silva, L.H.S. *et al.*, 2005 *Anuário do Instituto de Geociências* 28(1): 92-100. [in Portuguese]

16. Vasconcelos, C. *et al.*, 2006 *Sedimentary Geology* 185: 175-183.

17. Feder, F. *et al*., 2013. *Advances in Microbiology* 3: 47-54.

18. Guiry, M.D. and Guiry, G.M. 2016. AlgaeBase. World-wide electronic publication, National University of Ireland, Galway. http://www.algaebase.org; searched on 18 March 2016.

19. Komárek, J. *et al.*, 2014. *Preslia*, 86: 295-335.
