# Supplementary material for: Cyanobacterial Diversity in Microbial Mats from the Hypersaline Lagoon System of Araruama, Brazil: An In-depth Polyphasic Study
Source: Front Microbiol. 2017 Jun 30;8:1233. doi: 10.3389/fmicb.2017.01233 (PMC5492833; doi:10.3389/fmicb.2017.01233)
Supplement: Supplementary file 14 [file Table3.PDF]

Supplementary Material

Cyanobacterial diversity in microbial mats from the hypersaline coastal lagoon system of Araruama, Brazil: an in-depth polyphasic approach

Vitor Ramos[1,2], Raquel Castelo-Branco[1], Pedro Leão[1], Joana Martins[1,2], Sinda Carvalhal-Gomes[3], Frederico Sobrinho da Silva[3], João Graciano Mendonça Filho[3], Vitor Vasconcelos[1,2,*]

1 Faculty of Sciences, University of Porto, Porto, Portugal

2 Interdisciplinary Centre of Marine and Environmental Research (CIIMAR/CIMAR), University of Porto, Matosinhos, Portugal

3 Palynofacies & Organic Facies Laboratory, Department of Geology, Federal University of Rio de Janeiro, Brazil

*** Correspondence:** Vitor Vasconcelos: vmvascon@fc.up.pt

Supplementary Table S3. Percentage of pyrosequencing reads, by sample, included in each phylotype identified in the phylogenetic tree. Only clusters that include sequences derived from this study (i.e. obtained from 454-pyrosequencing, isolates or DGGE bands) were considered to distinguish phylotypes (see also Supplementary Image S2). Phylotypes with cells highlighted in blue indicate presence or absence of 454-read sequences in all three samples. Dark gray cells highlight the higher value for each sample. Light gray cells highlight values >4%.

Phylotypes	EB1	EB2	EB3
A	-	84,65%	3,08%
B	13,18%	-	-
C	22,86%	-	-
D	-	0,09%	0,07%
E	-	-	-
F	-	5,27%	-
G	-	2,18%	-
H	0,12%	-	-
I	0,31%	-	-
J	-	-	90,03%
K	0,25%	-	-
L	1,59%	0,34%	1,43%
M	3,25%	-	-
N	1,78%	-	-
O	-	-	0,14%
P	1,00%	0,25%	-
Q	0,56%	1,53%	-
R	0,47%	0,12%	-
S	0,31%	-	-
T	1,09%	-	-
U	1,56%	0,06%	-
V	5,03%	0,18%	-
W	1,25%	0,06%	1,26%
X	3,03%	-	-
Y	0,94%	-	-

Z	0,12%	-	-
AA	0,12%	-	-
AB	0,09%	-	-
AC	7,93%	0,18%	
AD	-	-	0,25%
AE	10,87%	0,12%	0,07%
AF	0,09%	-	-
AG	1,59%	-	-
AH	0,19%	0,06%	-
AI	0,19%	-	-
AJ	0,09%	-	-
AL	3,72%	-	-
AM	12,99%	2,14%	1,69%
**loner OTUs**			
EB1_33_V3Z02DFMNB	2,34%	-	-
EB1_39_V3Z02ERQP0	0,22%	-	-
EB1_77_V3Z02D1AW0	0,19%	-	-
EB1_75_V3Z02DA6N6	0,16%	-	-
EB1_70_V3Z02DPWRC	0,12%	-	-
EB2_2_V3Z04J04BU	-	0,09%	-
EB2_15_V3Z04I8TO7	-	0,09%	-
